# Fault-controlled reservoir compartmentalization as a control on CO_2_ storage potential in Jurassic Safa reservoirs, Obaiyed Field, Western Desert, Egypt

**DOI:** 10.1038/s41598-026-63564-2

**Published:** 2026-08-01

**Authors:** Walaa A. Ali, Abdelrazek Mohamed, Sherif Farouk

**Affiliations:** 1 Petroleum Geology Department, Faculty of Petroleum and Mining Sciences, Matrouh University, Matrouh, 51511, Egypt; 2 Exploration Department, Egyptian Petroleum Research Institute (EPRI), Cairo, 11727 Egypt

**Keywords:** Carbon capture and storage, Jurassic reservoirs, Fault compartmentalization, Tight sandstone reservoir, 3D static geological modeling, Shushan basin, Western Desert, Egypt, Energy science and technology, Solid Earth sciences

## Abstract

Geological carbon storage in mature hydrocarbon fields requires careful evaluation of reservoir architecture, fault-controlled compartmentalization, and sealing integrity to ensure long-term containment of injected CO_2_. This study presents an integrated geological assessment of the CO_2_ storage potential of the Jurassic Safa reservoirs in the Obaiyed Field, located within the Shushan Basin of Egypt’s Western Desert. The primary objective is to evaluate how structural segmentation and reservoir heterogeneity influence storage capacity and containment behavior in a deeply buried tight sandstone system. The study integrates seismic interpretation, well-log–based petrophysical analysis, core-derived permeability measurements, fault seal evaluation, and three-dimensional static geological modeling to characterize reservoir geometry, petrophysical variability, and structural compartmentalization. Results indicate that the Lower Safa Member represents a heterogeneous tight sandstone reservoir with effective porosity ranging approximately from 8 to 18% and permeability values spanning more than one order of magnitude. Porosity–permeability relationships derived from core measurements confirm significant reservoir heterogeneity and limited flow capacity typical of deeply buried clastic systems. Structural interpretation reveals pronounced fault-bounded compartmentalization that restricts lateral connectivity and promotes pressure partitioning within individual reservoir segments. Although storage capacity within individual compartments is modest, the cumulative storage potential of multiple structurally isolated compartments may provide a substantially larger field-scale storage resource. These findings highlight the potential of structurally segmented Jurassic reservoirs in the Western Desert to serve as viable candidates for geological CO_2_ storage and demonstrate the suitability of integrated geological screening for evaluating structurally compartmentalized reservoirs as potential CO_2_ storage sites.

**Supplementary Information:**

The online version contains supplementary material available at 10.1038/s41598-026-63564-2.

## Introduction

Geological carbon storage in subsurface reservoirs has become one of the key strategies proposed for mitigating anthropogenic carbon dioxide emissions and supporting the global transition toward low-carbon energy systems. Among the different storage options, depleted hydrocarbon reservoirs represent particularly attractive targets because their subsurface architecture, reservoir properties, and sealing systems have been extensively characterized through decades of exploration and production activities. In many cases, the long-term retention of hydrocarbons within these systems provides direct geological evidence of effective containment over geological timescales. Consequently, mature hydrocarbon provinces are increasingly being reassessed as potential CO_2_ storage sites where reservoir quality, structural closure, and caprock integrity can be reliably constrained within an integrated geological framework^1–3^.

In structurally complex sedimentary basins, however, reservoir compartmentalization and fault-controlled heterogeneity strongly influence fluid migration, pressure communication, and storage performance. Although structural segmentation often poses challenges for hydrocarbon production due to reduced reservoir connectivity, these same geological characteristics may offer advantages for CO_2_ storage by limiting plume migration and promoting pressure partitioning within discrete reservoir compartments. Understanding how reservoir architecture, heterogeneity, and fault-sealing behavior interact to control storage performance is therefore a critical step in evaluating CCS potential in mature hydrocarbon provinces. Integrated geological workflows combining seismic interpretation, petrophysical characterization, and three-dimensional reservoir modeling provide an effective framework for investigating these controls and identifying reservoirs capable of secure long-term CO_2_ storage^2,4,5^.

Within this context, Egypt’s Western Desert represents one of the most extensively explored sedimentary provinces in North Africa. Decades of hydrocarbon exploration have resulted in dense well coverage, extensive seismic datasets, and a well-constrained understanding of basin architecture and petroleum system evolution. These datasets provide a strong geological foundation for evaluating the region’s potential for geological CO_2_ storage, particularly within structurally complex but well-characterized reservoir systems^6–8^. Recent studies have therefore begun to explore the potential of depleted Jurassic and Cretaceous reservoirs across the Western Desert as candidate storage sites, particularly where structural closure and regionally extensive sealing formations are present^9–11^. Recent regional investigations of Cretaceous seals have been documented across the Northwestern Desert basins by^12^.

**Fig. 1:**
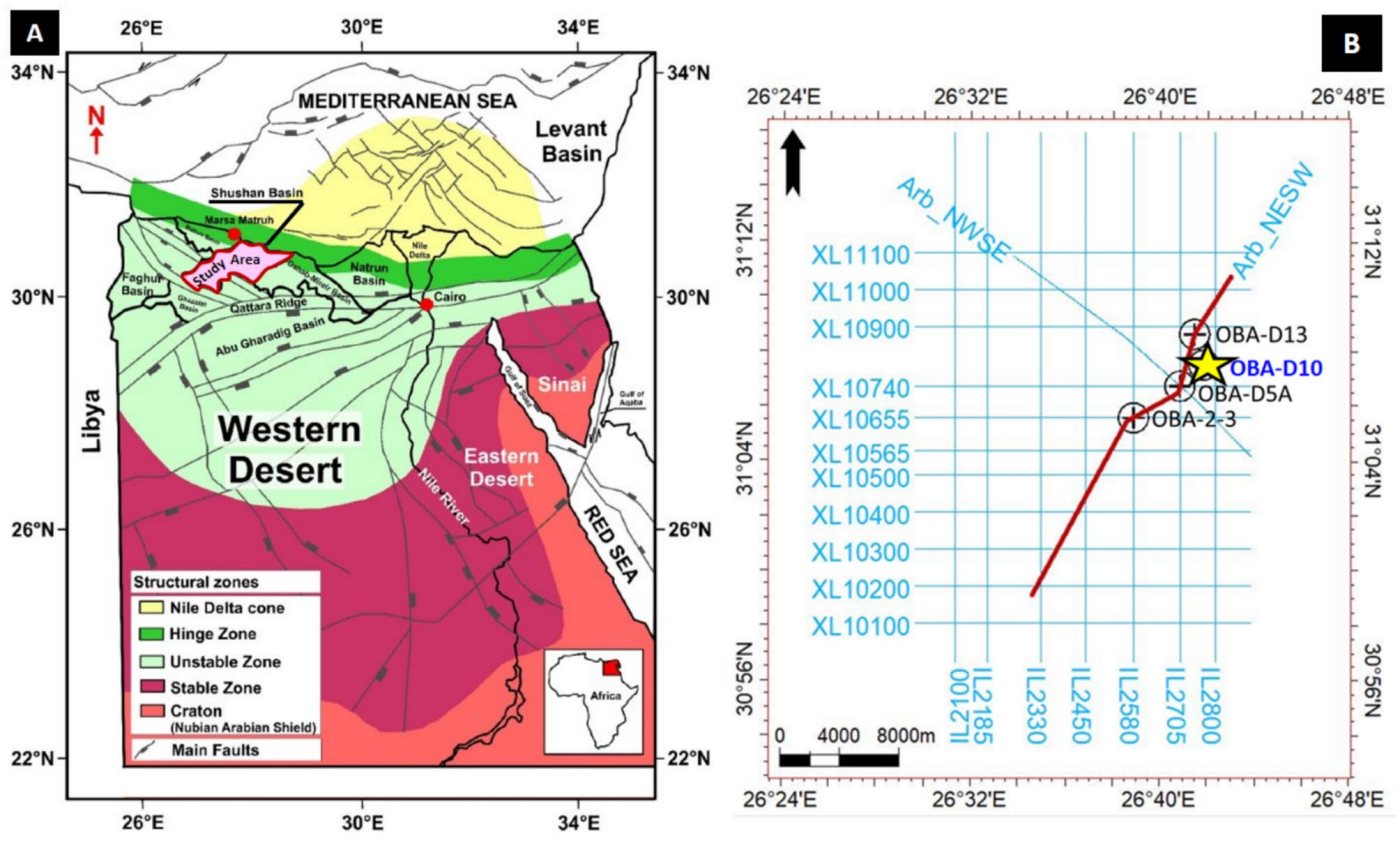
(**A**): A regional map of Egypt showing the location of the study area and the main structural zones (modified after^17^). (**B**): Location of the studied wells, interpreted seismic profiles, and the independent blind-validation well (OBA-D10) used for geological model verification.

The Obaiyed Field, located within the Shushan Basin, represents a fault-controlled Jurassic gas-condensate system developed within the Khatatba Formation (Fig. 1). Hydrocarbon accumulation within the field is governed by syn-rift depositional architecture, subsequent tectonic inversion, and pronounced fault-bounded compartmentalization^6,7,13^. While this structural segmentation has historically limited lateral reservoir connectivity and production performance, it may enhance the containment potential of CO_2_ storage systems by promoting pressure isolation and restricting large-scale plume migration within the reservoir.

Previous investigations in the Western Desert have largely focused on petroleum system evolution, depositional environments, and reservoir quality of the Jurassic successions^14–16^. However, few studies have integrated structural interpretation, reservoir heterogeneity analysis, and 3D geological modeling within a comprehensive CO_2_ storage assessment framework. As a result, the suitability of these systems for long-term CO_2_ storage has not yet been comprehensively evaluated.

**The objective** of this study is therefore to provide a geological screening assessment of CO_2_ storage potential within the Jurassic Safa reservoirs of the Obaiyed Field. The analysis focuses on the role of structural architecture, reservoir heterogeneity, and volumetric capacity estimation in a fault-segmented tight sandstone system. Rather than conducting detailed reservoir engineering simulations, the study emphasizes a geological framework integrating seismic interpretation, well-log petrophysical analysis, and three-dimensional static modeling to evaluate storage feasibility at the reservoir scale.

**The main contribution of this work** lies in reframing a depleted Jurassic tight-gas reservoir system—traditionally considered challenging for hydrocarbon development—as a potential candidate for geological CO_2_ storage. By extending conventional petroleum-system knowledge toward a storage-focused perspective, the study demonstrates how fault-controlled compartmentalization and stratigraphic heterogeneity influence storage capacity, injectivity behavior, and containment efficiency. Generally, the presence of multiple structurally isolated reservoir units suggests that combined storage potential across the field or basin may be significantly larger. The integrated workflow developed here provides integrated geological screening methodology for evaluating CCS potential in structurally complex tight clastic reservoirs within mature sedimentary basins.

## Geological, tectonic, and stratigraphic settings

**Fig. 2:**
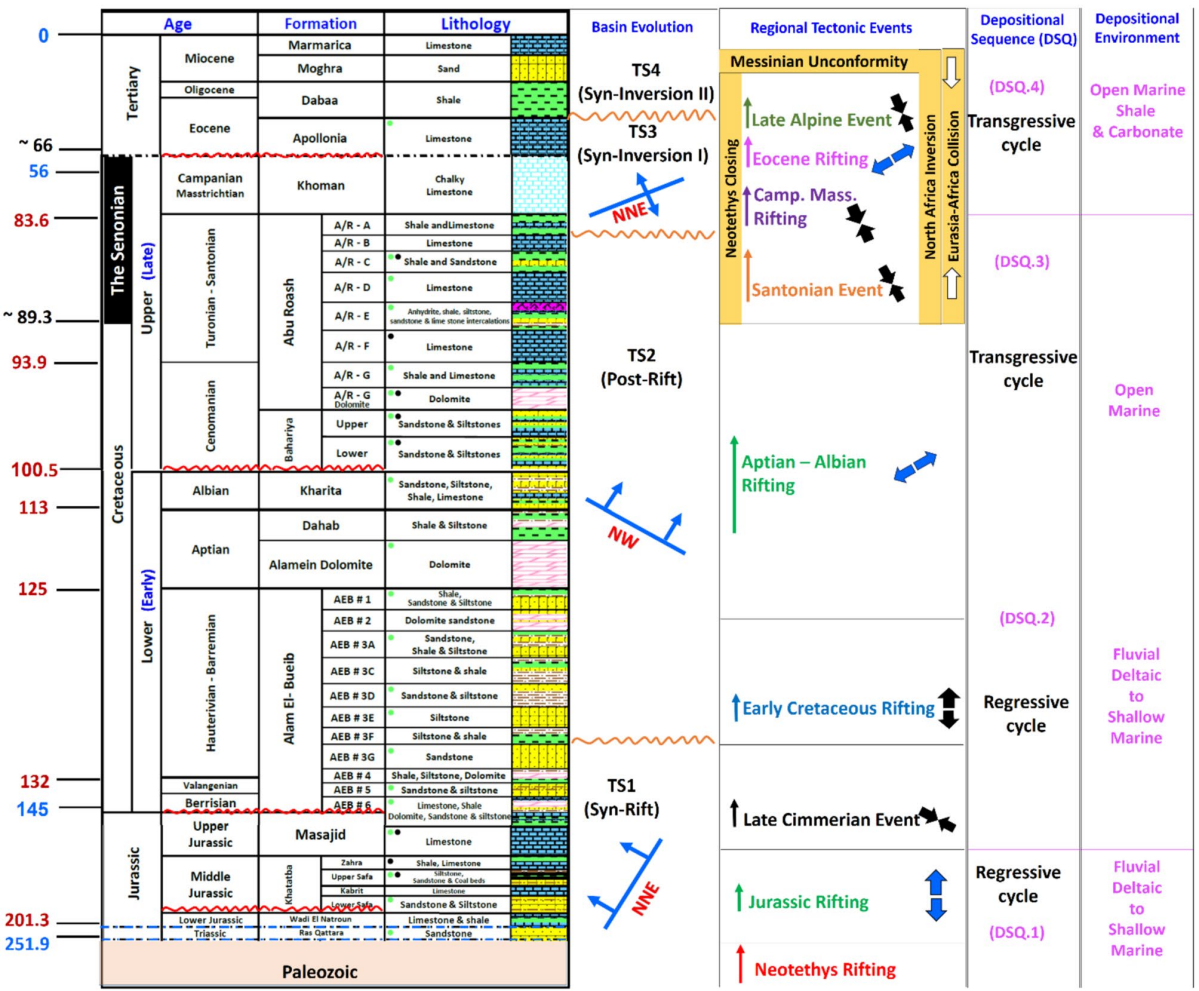
Composite stratigraphic column summarizing basin evolution, regional tectonic phases, and depositional environments of the Northwestern Desert, highlighting the tectono-stratigraphic framework relevant to reservoir development (Modified after^19^).

The Obaiyed Field is situated within the Shushan Basin of Egypt’s Northwestern Desert, where thick Mesozoic sedimentary successions overlie a structurally complex basement framework developed along the northern margin of the African Plate^7,18^. Prolonged subsidence and repeated tectonic reactivation generated a heterogeneous subsurface architecture in which fault geometry, stratigraphic subdivision, and reservoir compartmentalization exert first-order control on fluid distribution and pressure communication (Fig. 2). The basin structure reflects the superposition of early extensional deformation and later compressional reactivation. Jurassic–Early Cretaceous rifting established the primary fault framework, while Late Cretaceous to Early Eocene inversion modified trap geometry and enhanced structural segmentation within the Syrian Arc system^6,13^.

Stratigraphically, the Jurassic Khatatba Formation hosts the principal reservoir intervals in the Obaiyed Field. Storage potential is concentrated within the Safa members, particularly the Lower Safa Member, which consists of fluvio-estuarine to marginal-marine sandstones interbedded with finer-grained deposits, producing pronounced vertical and lateral heterogeneity^14,15^. Regionally developed Cretaceous shale and carbonate successions act as effective sealing units and have preserved hydrocarbon accumulations over geological timescales, providing independent evidence for long-term subsurface containment^8^. The combination of pronounced structural segmentation, stratigraphic variability, and demonstrated seal performance necessitates an integrated, multidisciplinary approach capable of resolving reservoir architecture, spatial property heterogeneity, and compartment-scale behavior. The following section therefore describes the datasets employed and the methodological framework adopted to characterize the Obaiyed Field and to evaluate its suitability for geological CO_2_ storage.

## Materials and methods

### Data

The subsurface dataset analyzed in this study was provided by Badr El-Deen Petroleum Company (BAPETCO) with formal authorization from the Egyptian General Petroleum Corporation (EGPC). The available data include wireline logs from three depleted wells (OBA-2–3, OBA-D5A, and OBA-D13) distributed across the Obaiyed Field. The log suite comprises gamma-ray, resistivity, caliper, neutron, density, photoelectric factor (PEF), density environmental correction, and acoustic measurements. Collectively, these datasets provide a robust basis for lithological discrimination, petrophysical characterization, and reservoir evaluation.

Reservoir pressure information derived from repeat formation tests (RFT) is available for all three wells and was used to assess pressure behavior, fluid distribution, and the degree of reservoir compartmentalization. Such pressure data are particularly important in tight, structurally segmented reservoirs, where limited hydraulic connectivity exerts a first-order control on both hydrocarbon performance and the suitability of formations for geological CO_2_ storage^20,21^.

The seismic dataset consists of two-dimensional (2D) seismic profiles acquired over the Obaiyed Field. These data were previously processed, including static and time-shift corrections, to ensure reliable time–depth relationships and consistent integration with well information. Although three-dimensional seismic coverage is not available, high-quality 2D seismic data, when constrained by dense well control, remain effective for delineating structural architecture and supporting reservoir-scale geological modeling in mature fields^6,22^ particularly when integrated with detailed well-log interpretation and static geological modeling.

### Methodological workflow

#### Seismic interpretation

**Fig. 3:**
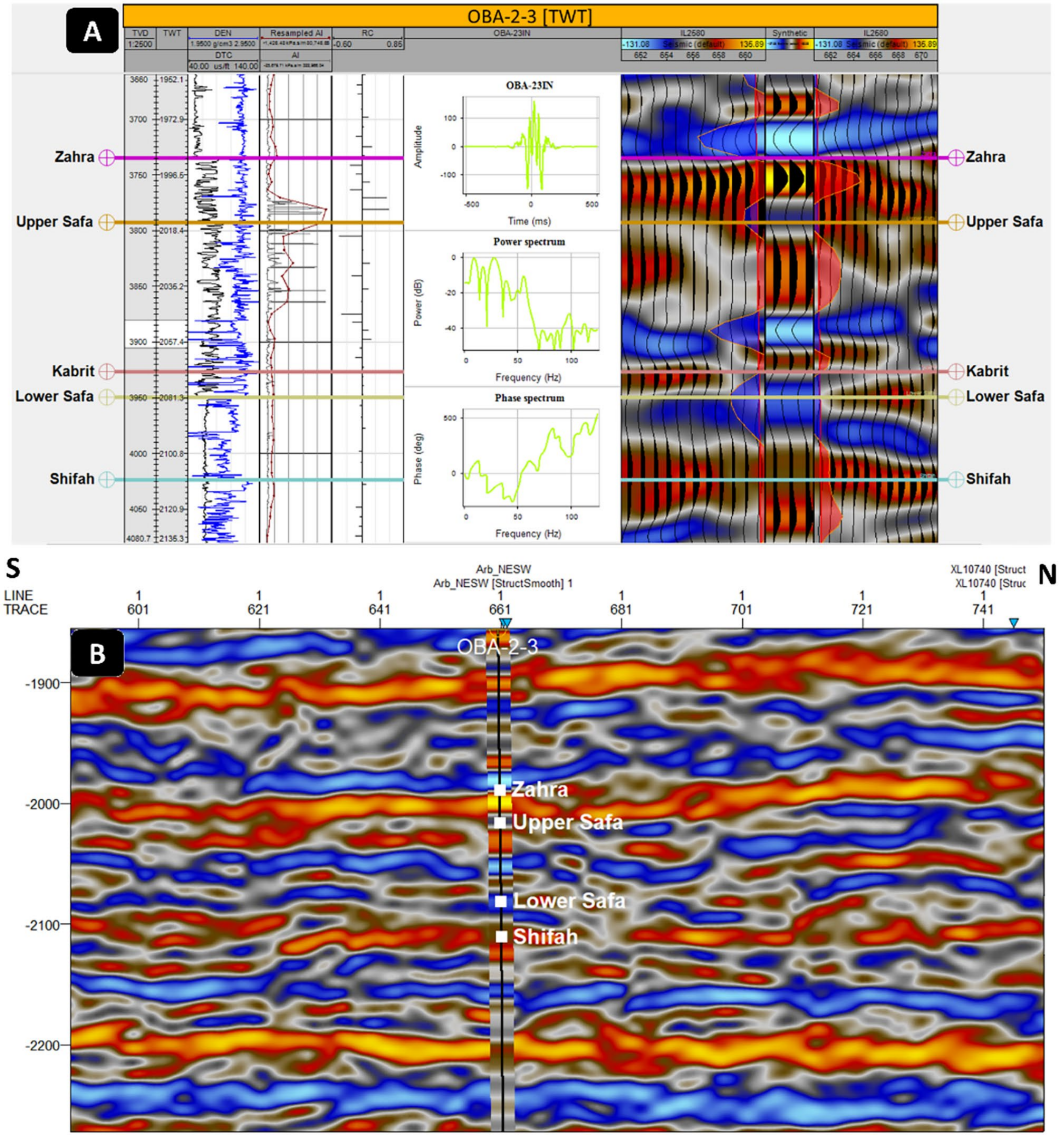
Seismic-to-well tie at well OBA-2–3 using a deterministic method and the extended white algorithm for time–depth calibration.

Seismic interpretation was undertaken to define the geometry, lateral extent, and structural configuration of the Jurassic reservoir intervals relevant to CO_2_ storage assessment. The interpretation emphasized the identification of key stratigraphic horizons and fault systems that govern reservoir architecture, structural compartmentalization, and trap development. All seismic interpretation and data integration were carried out using Petrel™ software. A seismic-to-well tie was established as an initial step to ensure reliable correlation between seismic reflections and formation tops identified in the wells (Fig. 3). Synthetic seismograms were generated from density and sonic logs and matched with adjacent seismic traces, allowing accurate horizon identification and time–depth calibration^23,24^. This procedure is particularly important in faulted and compartmentalized reservoirs, where relatively small depth uncertainties can have a disproportionate impact on volumetric calculations and the definition of structural closures.

**Fig. 4:**
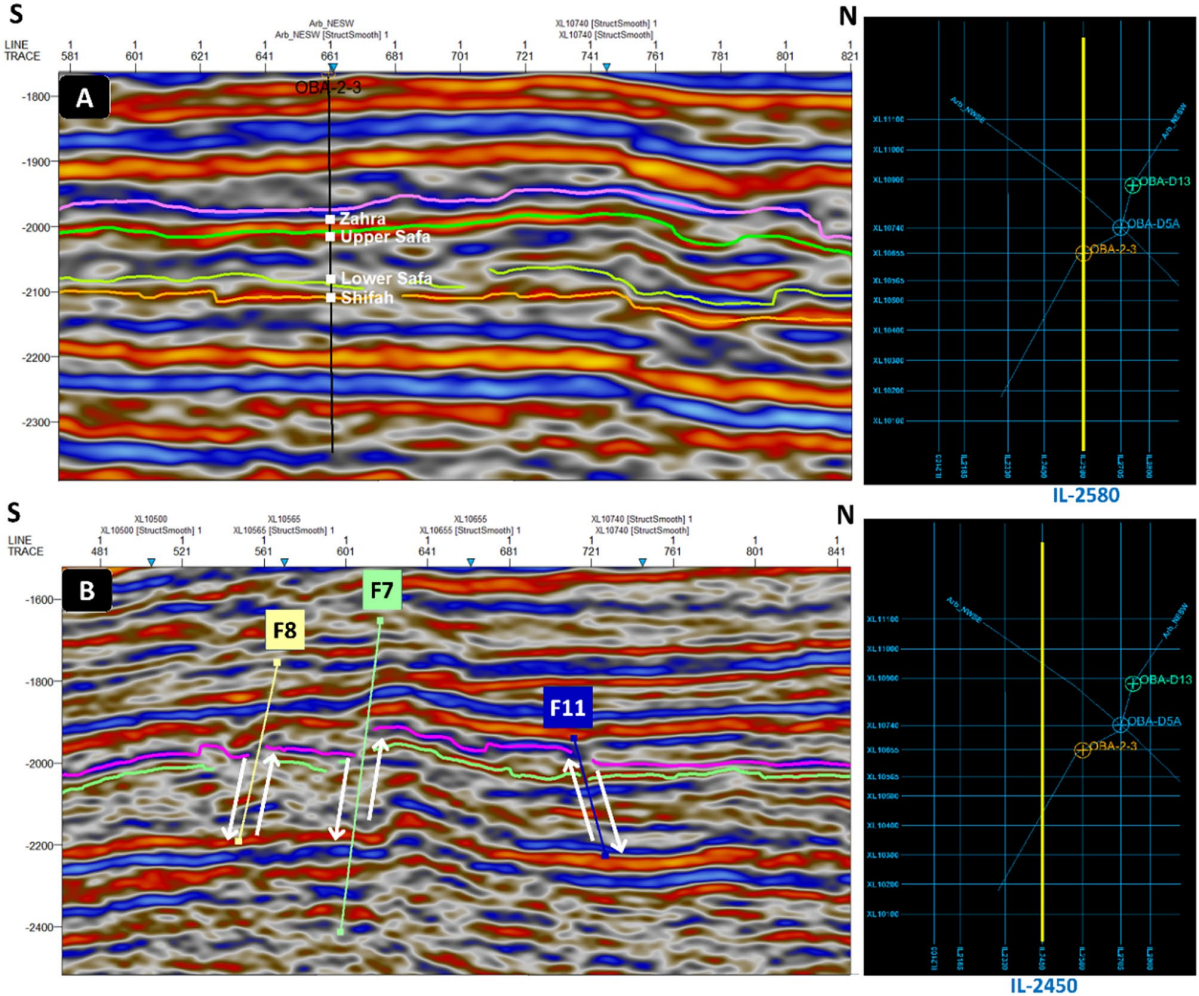
Seismic interpretation of the Obaiyed Field illustrating (**A**) key Jurassic horizon picks and (**B**) the interpreted fault framework that constrains structural architecture and reservoir compartmentalization.

After achieving satisfactory well ties, the principal Jurassic horizons were interpreted and tracked across the available seismic profiles (Fig. 4A). These interpretations were used to construct two-way travel time (TWT) maps, which were subsequently converted to depth using velocity functions calibrated to well data. Fault interpretation in (Fig. 4B) additionally constrained the geometry of fault-bounded compartments incorporated into the subsequent 3D geological model^4,5^. The resulting structural interpretation formed the basis for subsequent three-dimensional structural modeling and reservoir characterization.

#### Petrophysical analysis

Petrophysical evaluation of the Jurassic reservoir intervals was carried out using digital wireline log data from the three studied wells. The analysis incorporated gamma-ray, resistivity, neutron, density, and sonic logs to estimate lithology, shale volume, porosity, and water saturation. Where available, core-derived measurements were used to calibrate and validate log-based interpretations. All petrophysical analyses were performed using Techlog™^25–27^, with particular emphasis on the Quanti Elan module, which allows multi-mineral lithology modeling in heterogeneous clastic reservoirs. Prior to interpretation, the log data were subjected to quality control procedures, including depth matching, environmental corrections, and the removal of anomalous or unreliable measurements.

Lithological interpretation was constrained through integrated log responses within the Quanti Elan framework, incorporating quartz-rich sandstone, shale, and pore-fluid components consistent with the established lithological characteristics of the Lower Safa Member^14,15^. Shale volume was estimated using both the gamma-ray index method and neutron–density crossplot analysis. To minimize uncertainty, the lower shale volume obtained from these approaches was adopted for subsequent calculations^28,29^.

Water saturation was computed using the Indonesia saturation model, which is well suited for shaly sand reservoirs and has been widely applied to Jurassic clastic systems in the Northwestern Desert^30,31^. Effective porosity was derived by correcting total porosity for shale-bound water, yielding a more representative estimate of the pore volume available for fluid storage and migration.

#### Static geological modeling

Three-dimensional static geological modeling was applied to integrate seismic interpretation, well control, and petrophysical results into a unified representation of reservoir architecture and heterogeneity. The modeling workflow followed established geostatistical principles and comprised successive stages of structural modeling, facies modeling, and petrophysical property modeling^32,33^.

The three-dimensional geological model was discretized into **116 × 118 × 1080 grid cells**, with average horizontal cell dimensions of approximately **301 × 303 m**. Vertical layering was defined proportionally according to reservoir thickness, resulting in 400 layers for the Shifah Formation, 300 layers for the Lower Safa Member, 200 layers for the Upper Safa Member, 60 layers for the Kabrit Member, and 120 layers for the Zahra Member. Grid configuration is presented in supplementary (Table S1).

#### Structural modeling

Structural modeling was constructed using interpreted seismic horizons and fault geometries, with calibration provided by well tops. Major fault systems and stratigraphic surfaces were explicitly incorporated to define fault-bounded reservoir compartments within the Jurassic intervals. The reservoirs were subdivided into stratigraphic zones and discretized into layers of near-uniform thickness to adequately capture vertical variability and preserve stratigraphic continuity^34^. Fault interpretation emphasized the identification of major displacement zones, relay structures, and fault intersections that exert a strong influence on reservoir connectivity and pressure communication. These structural elements were fully integrated into the geological framework used for volumetric assessment and static modeling, reflecting their central role in controlling fluid migration, pressure evolution, and containment behavior in tight, fault-segmented reservoirs. The resulting structural model provides a quantitative foundation for subsequent reservoir analysis and CCS-focused evaluation of injectivity and storage security.

#### Well-log upscaling

Prior to property modeling, wireline log data were upscaled to the three-dimensional geological grid to ensure consistency between well-scale measurements and grid resolution. Petrophysical properties were vertically averaged within each grid cell using thickness-weighted averaging, preserving the contribution of thin beds while reducing scale mismatch. The structural framework was discretized into 120 layers for the Zhara Formation, 200 layers for the Upper Safa Member, 60 layers for the Kabrit Formation, 300 layers for the Lower Safa Member, and 400 layers for the Paleozoic Shifa Formation. The resulting upscaled properties were subsequently used for facies and petrophysical modeling throughout the 3D geological model.

#### Facies modeling

Facies modeling aimed to represent the spatial distribution of reservoir and non-reservoir lithofacies using log-derived facies classifications conditioned to the structural framework. Given the limited number of wells, stochastic modeling approaches were employed to account for geological uncertainty away from direct well control and to generate realistic lateral facies transitions^33^. Variogram parameters used for stochastic facies modeling are presented in supplementary (Table S2).

#### Property modeling

Petrophysical modeling was then performed to populate the three-dimensional grid with effective porosity and fluid saturation values. Property distribution was constrained by the modeled facies architecture and conditioned to well data using geostatistical algorithms, ensuring that spatial continuity was honored while preserving observed heterogeneity^3,34^.

Property modeling was performed using Sequential Indicator Simulation (SIS). A spherical variogram model was adopted with a nugget of **0.0001**, sill of **0.9999**, horizontal ranges of **11,818 m**, and formation-specific vertical ranges of **0.7–2.6 m**, depending on the modeled stratigraphic interval.

Mutually, these modeling stages provide an explicit representation of reservoir architecture, spatial heterogeneity, and fault-controlled compartmentalization. Such static geological models form a critical basis for subsequent dynamic simulations and risk-oriented evaluations of CO_2_ injection and long-term storage performance^3,20^.

#### Fault seal and transmissibility analysis

To improve the assessment of fault-controlled fluid flow and containment behavior, a semi-quantitative fault seal analysis was conducted using the three-dimensional structural model. In addition to lithological **juxtaposition analysis**, several fault-related parameters were evaluated, including fault gouge ratio (FGR), fault permeability prediction, fault thickness–displacement relationships, and effective cross-fault transmissibility. **Fault thickness** was estimated from fault displacement and fault-rock property calculations and subsequently incorporated into the fault permeability and transmissibility analyses. **Fault gouge ratio** was used as a proxy for clay content within fault zones and its impact on sealing behavior, following established approaches for predicting fault seal capacity in clastic reservoirs^35^. **Fault permeability** was estimated by integrating core-derived porosity and permeability data with fault rock properties, allowing prediction of permeability reduction within fault zones^36^. **Cross-fault transmissibility multipliers** were calculated to quantify the degree of hydraulic communication across faults, reflecting the combined effects of fault rock properties and stratigraphic juxtaposition^37^. These parameters were used to classify faults into sealing, partially sealing (baffle), and potentially transmissive categories, providing a more robust basis for evaluating reservoir compartmentalization and CO_2_ containment behavior in structurally complex systems^4^.

#### Methodology for CO_2_ storage capacity estimation in the Obaiyed Field

The methodology adopted to estimate CO_2_ storage capacity in the Obaiyed Field is based on a volumetric approach tailored to tight, structurally compartmentalized reservoirs. This approach is consistent with widely applied CCS assessment frameworks developed for mature hydrocarbon provinces and provides a robust first-order evaluation of storage potential by integrating reservoir geometry, petrophysical properties, and storage efficiency constraints derived from geological modeling^1,20,38^ .CO_2_ storage capacity was calculated using petrophysical parameters obtained from well-log interpretation and constrained by the three-dimensional static geological model. The key input parameters include shale volume (Vsh), total porosity (Φ_T), effective porosity (Φ_eff_), water saturation (S_w_), and net reservoir thickness. This workflow follows methodologies previously applied to depleted reservoirs in Egypt’s Western Desert, where tight reservoir conditions and structural segmentation strongly influence storage performance^9,39^. Reservoir intervals identified as non-reservoir or waste zones based on thickness criteria and petrophysical cutoffs were excluded to ensure conservative and geologically realistic capacity estimates.

The mass of stored CO_2_ (M_CO_2_) was estimated using the volumetric relationship:

1 $${\mathrm{MCO}}_{{2}} = {\mathrm{Vpv}} \times \left( {1 - {\mathrm{Swi}}} \right) \times {\mathrm{B}} \times \rho {\mathrm{CO}}_{{2}} \times {\mathrm{E}}$$

where Vpv, is the pore volume available for storage, Swi is the irreducible water saturation, B is the formation volume factor accounting for in-situ conditions, ρCO_2_​​ is the density of CO_2_ at reservoir pressure and temperature, and E is the storage efficiency factor reflecting geological and operational constraints. The method applied in this study follows internationally recognized screening frameworks used for preliminary CCS capacity estimation prior to dynamic simulation^38,40^.

All parameters were handled using SI units to ensure dimensional consistency throughout the calculations. CO_2_ density values were selected according to reservoir depth and pressure–temperature conditions, assuming supercritical CO_2_ behavior under injection conditions^20^. An efficiency factor “E” of **0.8** represents a site-specific assumption to characterize compartment-scale storage within structurally confined reservoirs. This value provides a first-order estimate and does not account for pressure constraints, injectivity limitations, or dynamic plume evolution. Consequently, the factor (E = 0.8) is intended for compartment-scale geological screening under structurally confined reservoir conditions and should not be interpreted as a universally applicable value for all geological CO_2_ storage projects^9,10^.

Storage capacity calculations were carried out independently for each well (OBA-2–3, OBA-D5A, and OBA-D13) and for individual stratigraphic units within the Upper and Lower Safa members. This unit-based approach allows the effects of stratigraphic heterogeneity and fault-controlled compartmentalization on CO_2_ storage capacity to be explicitly evaluated, rather than relying on field-averaged parameters. Although volumetric calculations provide a first-order estimate of theoretical storage capacity, the effective volume of injectable CO_2_ in tight and compartmentalized reservoirs is ultimately constrained by pressure limits and injectivity considerations. Accordingly, the volumes reported here represent storage-limited estimates that define a geological framework for subsequent pressure-constrained evaluation and risk assessment, consistent with established best practices in CCS site screening^3,20^.

## Results

### Reservoir petrophysical characteristics

#### Lithology interpretation

**Fig. 5:**
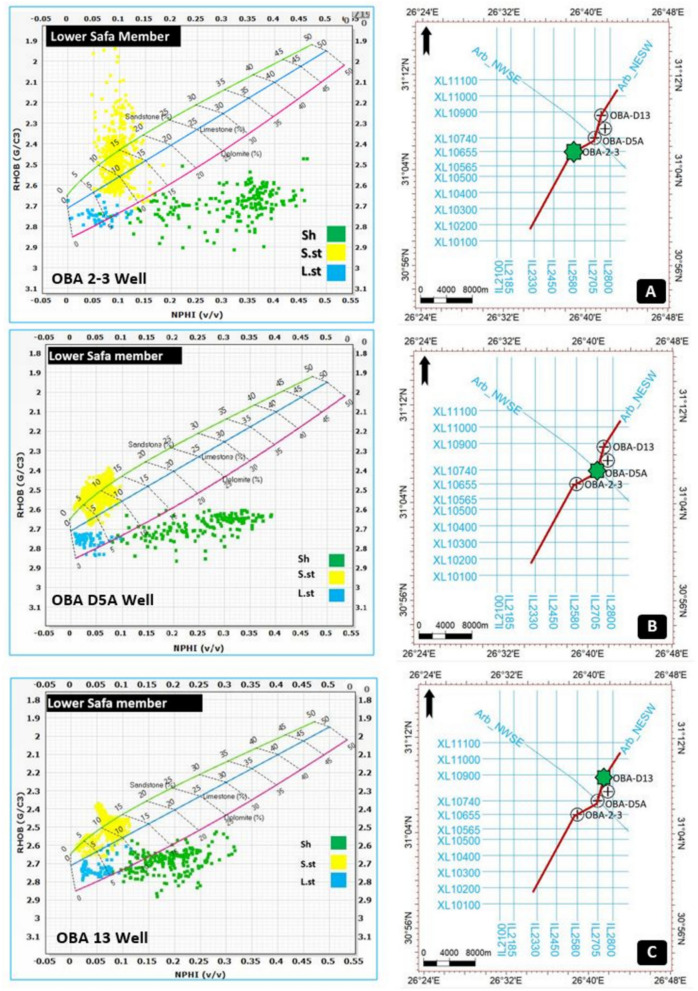
The neutron-density cross plots illustrating lithological discrimination of (**A**):OBA2-3 well; (**B**): OBA-D5 well; (**C**): OBA-13 well (Generated in Techlog, 2018 software).

Petrophysical evaluation of the Jurassic Safa reservoirs indicates marked vertical and lateral heterogeneity between the Upper and Lower Safa members across the studied wells (OBA-2–3, OBA-D5A, and OBA-D13). Lithological interpretation was performed using Techlog™ 2018, integrating wireline log responses with neutron–density crossplots, shows that the reservoir intervals are predominantly composed of quartz-rich sandstones interbedded with variable proportions of shale. This lithological assemblage is consistent with previously documented fluvio-estuarine to marginal-marine depositional systems of the Khatatba Formation^14,15^. Following lithology determination using **Quanti Elan** multi-mineral model, (Fig. 5) an independent validation was performed using **the** Schlumberger POR-15 deterministic crossplot, providing additional confidence in the lithological interpretation.

**Fig. 6:**
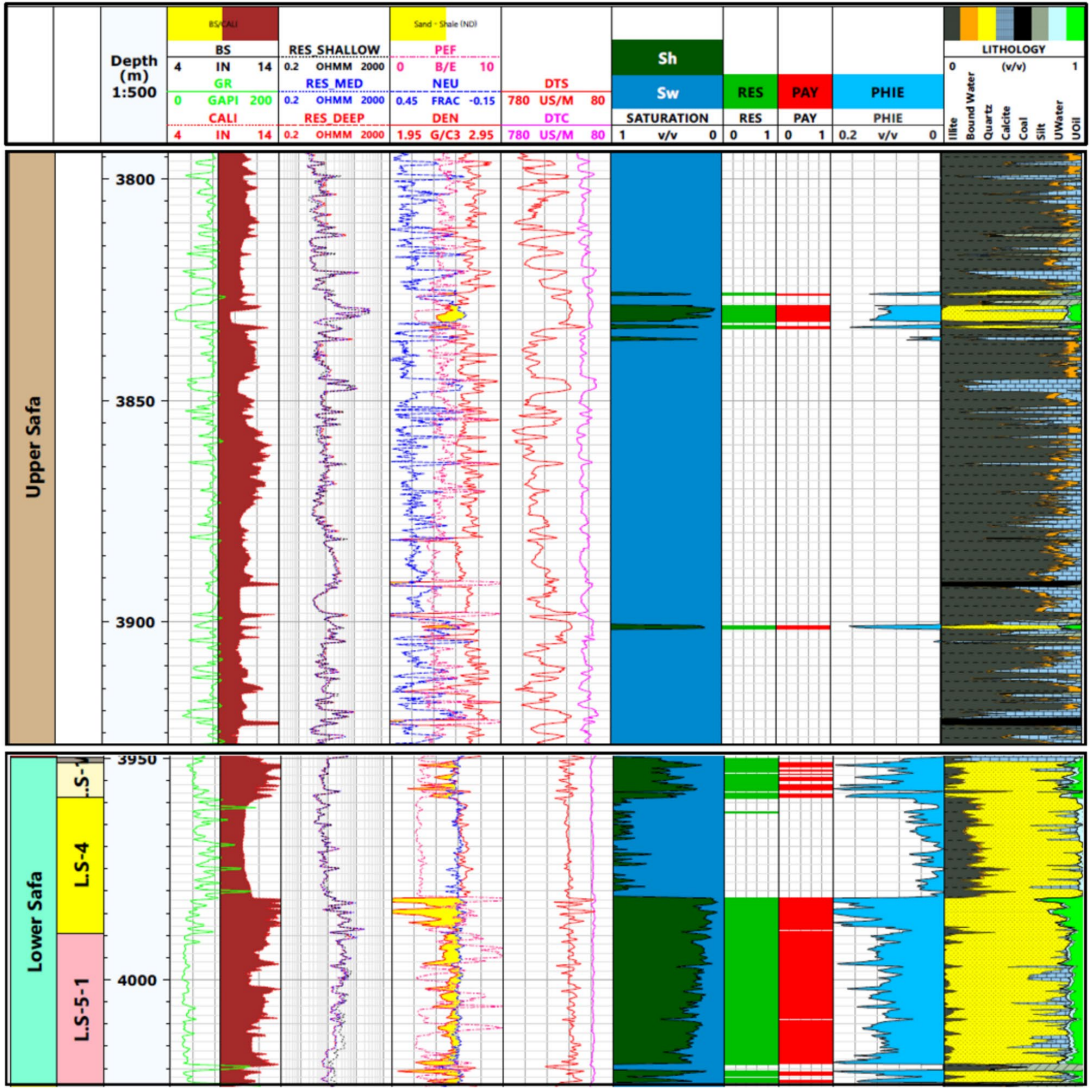
The lithosaturation panel of the **OBA-2–3** well illustrates Upper and Lower Safa petrophysical analysis and lithology interpretation by Quanti Elan.

**Fig. 7:**
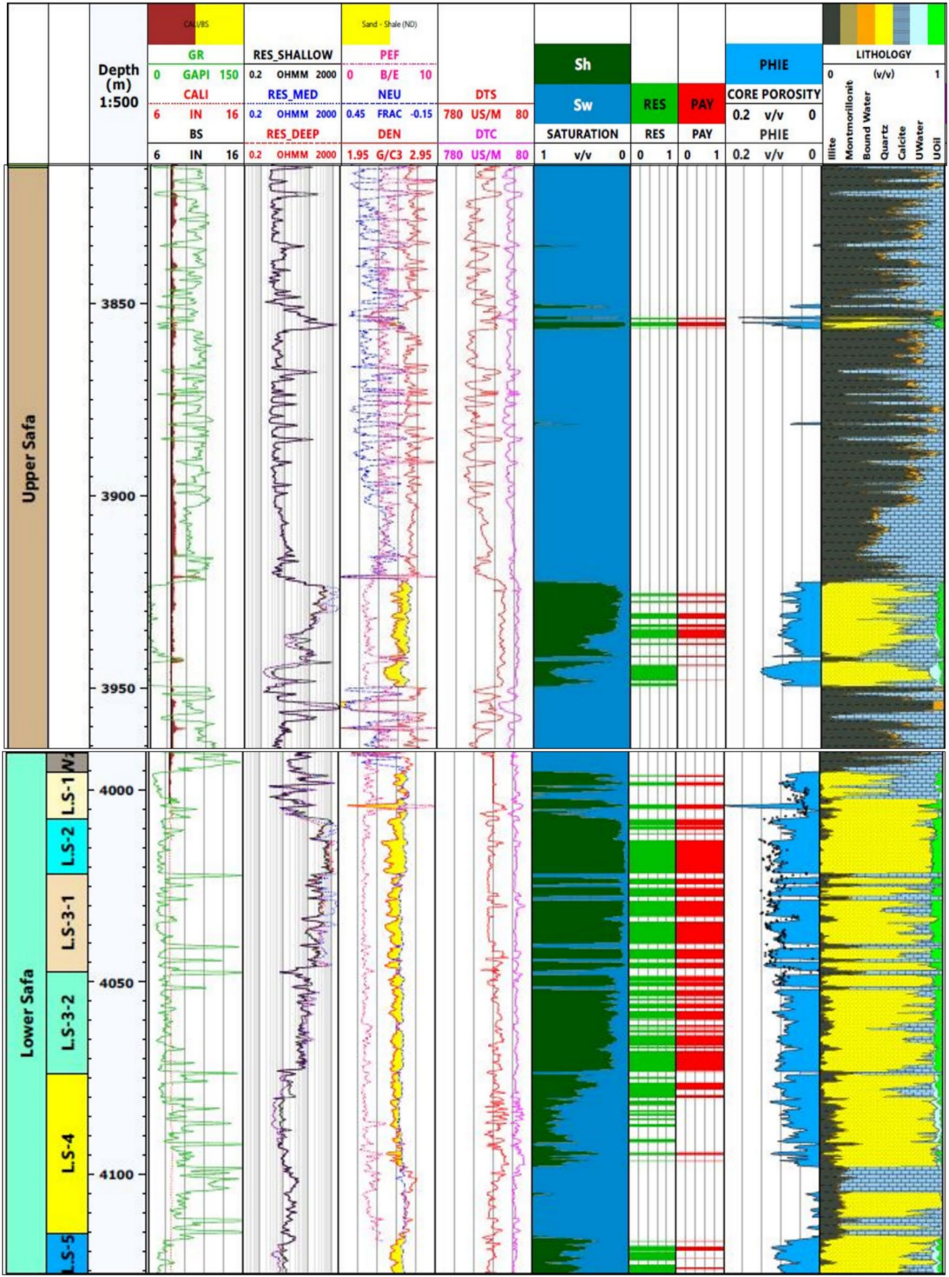
The lithosaturation panel of the **OBA-D5** well illustrates upper and lower Safa petrophysical analysis and lithology interpretation by Quanti Elan.

**Fig. 8:**
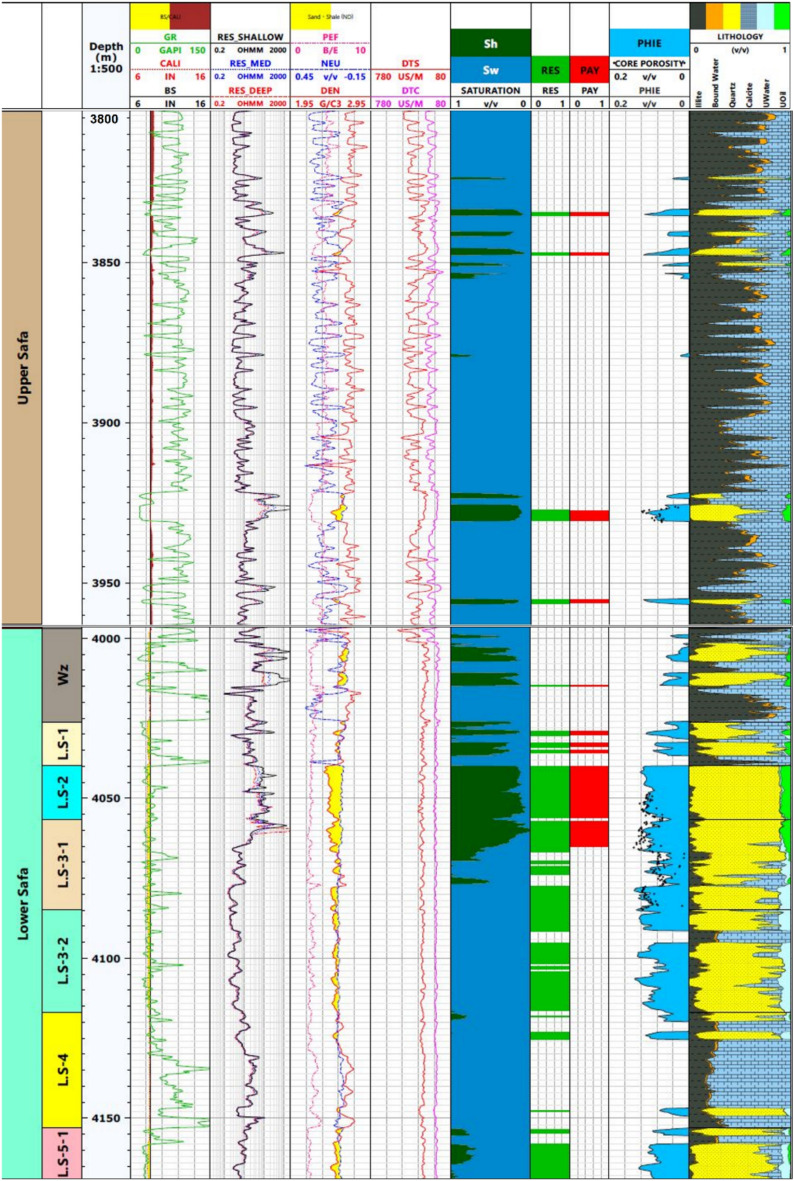
The lithosaturation panel of the **OBA-D13** well illustrates upper and lower Safa petrophysical analysis and lithology interpretation by Quanti Elan.

Lithology–saturation crossplots, were subsequently used as a robust tool for reservoir evaluation and decision-making regarding interval classification and testing potential. CPI crossplots constructed for the Lower Safa Formation in the three wells integrate multiple petrophysical parameters, including shale volume (Vsh), effective porosity (Φ_eff), net pay, gross thickness, and hydrocarbon saturation. These parameters were computed and visually represented in (Figs. 6, 7, and 8). Summary of the calculated petrophysical results for wells OBA-2–3, OBA-D5, and OBA-D13 is presented in Tables 1, 2, and 3, respectively.

**Table 1:** Log-derived petrophysical properties of the Upper and Lower Safa members in well OBA-2–3, Obaiyed Field, including porosity, shale volume, water saturation, and net reservoir thickness.

Well-OBA 2–3	Upper Safa	Lower Safa			
Zones	U.Safa	L.Safa	L.Safa UNIT_1	L.Safa UNIT_4	L.Safa UNIT_5-1
Top (m)	3793.56	3949.97	3951.15	3958.9	3989.59
Bottom (m)	3927.81	3951.15	3958.9	3989.56	4023.96
Gross thickness (m)	134.25	1.18	7.75	30.66	34.37
Net reservoir (m)	5.639	0.18	4.55	7.953	30.722
Net to gross	0.042	0.152	0.587	0.259	0.894
Av_shale volume (%)	22.7	12.1	9.3	18.7	4.7
Av_porosity (%)	11.3	10.7	14.7	17.9	11.9
Av_water saturation (%)	23.4	38.7	35.5	15.4	26.2

**Table 2:**
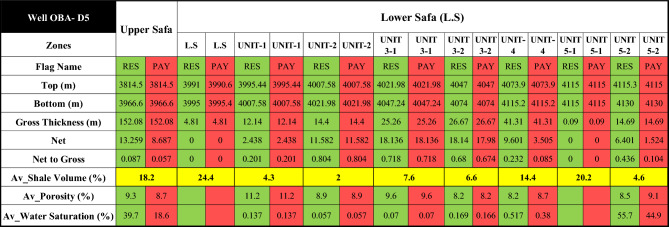
Summary of log-derived petrophysical parameters for the Upper and Lower Safa members in wells OBA-D5, Obaiyed Field.

**Table 3:**
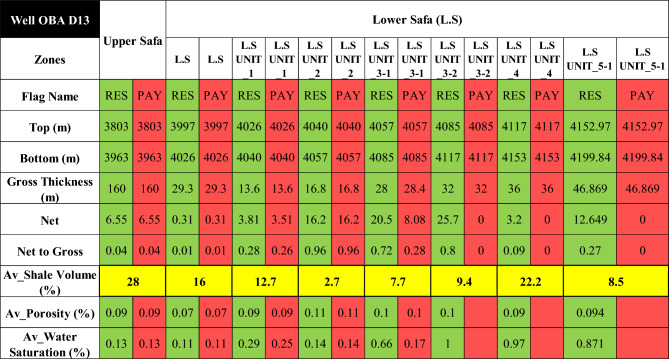
Summary of log-derived petrophysical parameters for the Upper and Lower Safa members in wells **OBA-D13**, Obaiyed Field.

**The Lower Safa Member** consistently exhibits more pronounced gross and net thickness than the Upper Safa Member across the three wells (Tables 1, 2 and 3). In wells OBA-D5 and OBA-D13, subdivision into multiple internal units (UNIT-1, UNIT-4, and UNIT-5–1) results in significant vertical stacking, indicating enhanced accommodation space and sustained sediment supply during deposition. Consequently, the Lower Safa provides increased effective storage volumes than the Upper Safa, representing an important advantage for both hydrocarbon accumulation and potential CO_2_ storage. **The Upper Safa Member** displays moderate and relatively uniform net-to-gross (NTG) values, whereas the Lower Safa Member shows better variability and locally higher NTG, particularly in UNIT-4 and UNIT-5–1 (Tables 2 and 3). Reduced NTG in some Lower Safa subunits reflects increased shale interbeds and stronger internal heterogeneity, indicating that the Upper Safa is more laterally uniform, while the Lower Safa exhibits heterogeneous but locally better-developed reservoir intervals.

#### Core analysis

Core measurements from wells penetrating the Lower Safa Member indicate permeability values ranging from approximately 0.03 to 25 mD and porosity values between ~ 8 and 18% (Table 4). These results confirm that the Lower Safa reservoir represents a moderately tight clastic system characterized by significant variability in reservoir quality. **In contrast**, core data from the underlying Paleozoic Shifa Formation show substantially lower permeability values, typically below 1 mD, with porosity ranging between ~ 6 and 10%. This marked contrast suggests limited vertical hydraulic communication between the two formations within the Jurassic reservoir system.

**Table 4:** Core-derived petrophysical properties.

Formation	Wells	Porosity (%)	Permeability (mD)
Lower Safa	Well-2	8–17	0.03–25
Lower Safa	Well-3	9–18	0.05–20
Shiffah	Well-1	6–10	0.01–3

#### Porosity distribution

The Upper Safa Member generally exhibits higher and more uniform effective porosity compared to the Lower Safa Member. In contrast, the Lower Safa displays a wider range of porosity values, indicating wider porosity variability than the Upper Safa Member. Additionally, facies vary with increasing burial depth, particularly in wells OBA-2–3 and OBA-D5A. These trends are consistent with regional observations from Jurassic tight-gas reservoirs in the Northwestern Desert, where porosity evolution is strongly controlled by depositional facies and burial-related diagenesis^14,15^.

#### Permeability characteristics

Permeability distribution reflects pronounced heterogeneity within the Safa reservoirs. While the Upper Safa Member generally exhibits relatively higher and more laterally consistent permeability, the Lower Safa is characterized by lower and more variable permeability values, locally approaching tight-gas conditions, particularly within UNIT-1 and UNIT-4. These results indicate that the Upper Safa is hydraulically more conductive, whereas the Lower Safa exhibits restricted flow capacity and stronger compartmentalization.

#### Heterogeneity quantification

Coefficient of variation (CV):

The degree of reservoir heterogeneity was quantified using the coefficient of variation (CV), which describes the relative dispersion of permeability values:

2 $$CV = \frac{\sigma }{\mu }$$

where,

CV = coefficient of variation (dimensionless)

*σ*_k_ = standard deviation of permeability (mD)

*μ*_k_ = mean permeability (mD)

Dykstra–Parsons Coefficient (VDP):

Reservoir heterogeneity was further evaluated using the Dykstra–Parsons coefficient, which characterizes permeability variation based on cumulative distribution:

3 $$V_{DP} = \frac{{k_{50} - k_{84.1} }}{{k_{50} }}$$

where,

V_*DP*_ = Dykstra–Parsons coefficient (dimensionless).

*k*_*50*_ = median permeability (mD), corresponding to the 50th percentile.

*k*_*84.1*_ = permeability at the 84.1th percentile (mD), representing one standard deviation in a log-normal distribution.

The Dykstra–Parsons coefficient ranges from 0 (homogeneous reservoir) to values approaching 1 (highly heterogeneous reservoir). Intermediate values indicate moderate heterogeneity typical of clastic reservoirs. The highest degree of heterogeneity is observed in the Lower Safa interval of Well-3, where permeability variability reflects strong spatial differences in pore-throat structure and reservoir quality.

#### Reservoir heterogeneity

Core-derived permeability data indicate moderate-to-high heterogeneity within the Lower Safa Member. The coefficient of variation (CV) ranges between approximately 0.40 and 0.55 across the analyzed wells. While, Dykstra–Parsons coefficients (**VDP**), range between ~ 0.4 and 0.6 (Table 5). The calculated CV and VDP values consistently indicate heterogeneous flow behavior typical of tight clastic reservoirs. Such heterogeneity is expected to influence fluid migration, pressure propagation, and CO_2_ distribution during injection by promoting compartment-scale flow rather than uniform reservoir communication.

**Table 5:** Core-derived reservoir heterogeneity.

Formation	Well	Φ mean (%)	k mean (mD)	CV	VDP
Lower Safa	Well-2	~ 12	~ 4–6	0.40–0.45	0.5–0.6
Lower Safa	Well-3	~ 13	~ 5–7	0.50–0.55	0.5–0.6
Shiffah	Well-1	~ 7–8	~ 0.05–0.2	0.40–0.45	0.4–0.5

*Values are presented as representative ranges due to variability across wells.

#### Interpretation of reservoir quality

The Lower Safa Member exhibits moderate to high heterogeneity and higher permeability compared to the underlying Shifa Formation, indicating better reservoir quality and flow capacity. In contrast, the Shifa Formation represents a lower-permeability unit that likely acts as a basal hydraulic barrier, limiting vertical fluid migration. This contrast reinforces the interpretation that CO_2_ storage in the Obaiyed Field is controlled by compartmentalized flow behavior within the Lower Safa reservoir, with vertical containment supported by underlying low-permeability formations.

#### Porosity–permeability relationship and flow heterogeneity

**Fig. 9:**
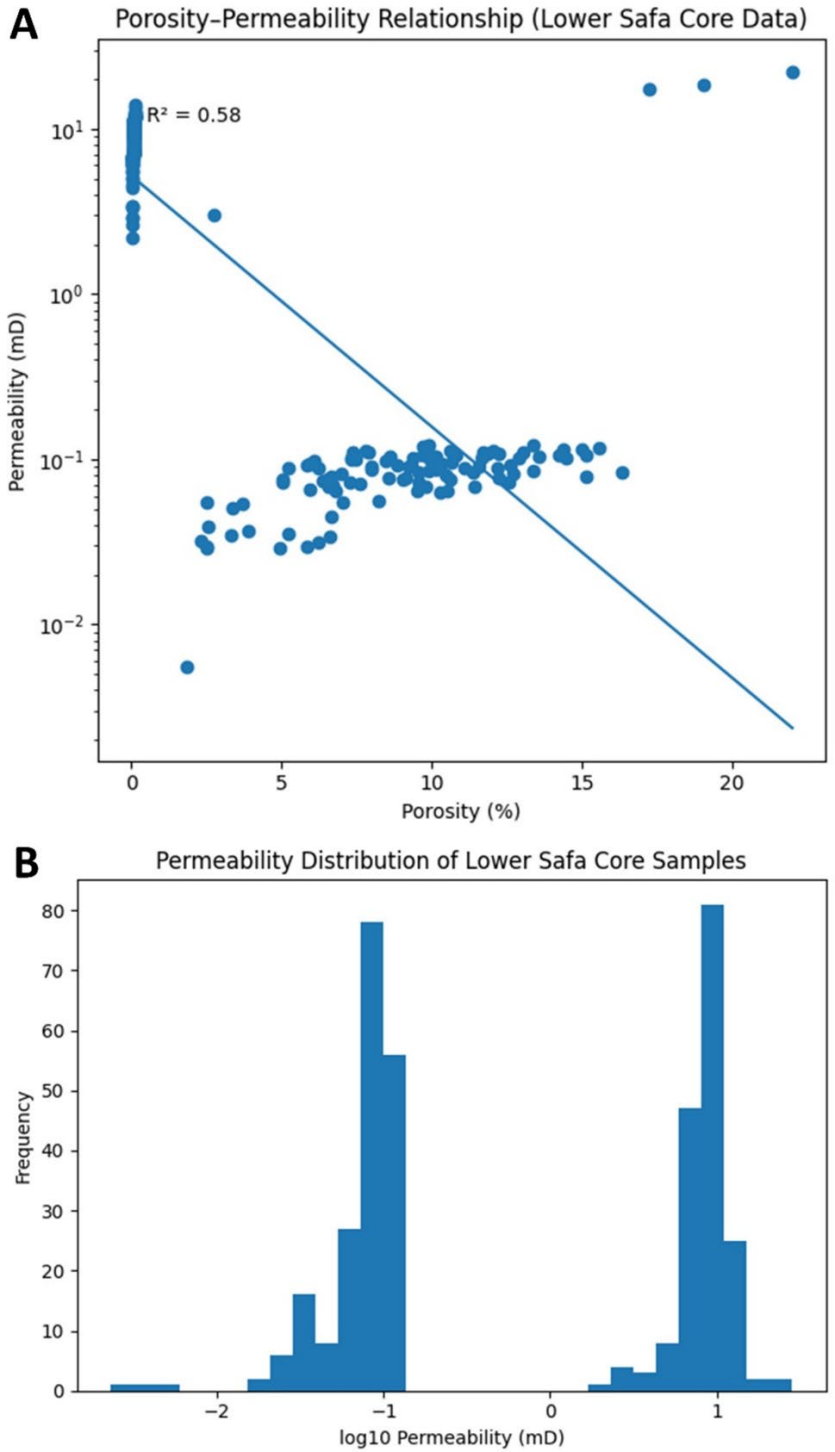
(**A**) Porosity–permeability crossplot for core samples from the lower Safa member of wells (OBA-D13, OBA-D5A) illustrating the heterogeneous flow capacity of the Jurassic sandstone reservoir. (**B**) Histogram showing the distribution of permeability values derived from core measurements of the Lower Safa Member in wells OBA-D5 and OBA-D13.

To evaluate reservoir flow capacity, the relationship between porosity and permeability was examined using core-derived measurements from the Lower Safa Member in wells OBA-D5A and OBA-D13 (Fig. 9A). The crossplot shows a broadly positive but highly scattered relationship, with permeability values spanning more than one order of magnitude for comparable porosity ranges. This dispersion indicates that permeability is not only controlled by porosity but is strongly influenced by variations in pore-throat geometry. The logarithmic distribution of permeability values reflects the tight nature of the reservoir and the influence of diagenetic modification on pore connectivity. This observed variability is consistent with the effects of burial-related diagenesis, including compaction and cementation, which modify pore connectivity and reduce effective flow pathways in deeply buried clastic reservoirs. Such behavior is widely reported in tight sandstone systems, where permeability is highly sensitive to small-scale pore structure changes rather than bulk porosity alone^43,44^.

The permeability distribution (Fig. 9B) further highlights this heterogeneity. Most values cluster within the low-permeability range typical of tight reservoirs, while a limited number of higher-permeability measurements indicate localized zones of enhanced pore connectivity. This bimodal-to-skewed distribution suggests that fluid flow within the Lower Safa Member is controlled by spatially variable permeability pathways rather than uniform matrix flow. From a CO_2_ storage perspective, this heterogeneity has two key implications. First, it constrains injectivity at the matrix scale, requiring pressure-limited injection strategies. Second, it promotes localized flow pathways that may influence plume geometry and pressure distribution within fault-bounded compartments. These results provide a quantitative basis for assessing the potential influence of permeability variability on injectivity and pressure-limited storage behavior.

#### Reservoir differentiation and storage implications

Petrophysical results indicate a clear contrast between the Upper and Lower Safa members. The Upper Safa generally exhibits higher water saturation and thinner but laterally more continuous intervals, whereas the Lower Safa is characterized by thicker pay zones with lower and more variable water saturation, particularly within structurally isolated compartments. Although the Upper Safa contributes limited effective storage capacity, represents a secondary storage interval influencing pressure distribution. In contrast, the Lower Safa Member represents the primary storage target due to its greater net thickness, effective pore volume, and structural segmentation. This reservoir-scale differentiation highlights the importance of unit-specific characterization when evaluating mature tight-gas systems for CCS applications.

**Fig. 10:**
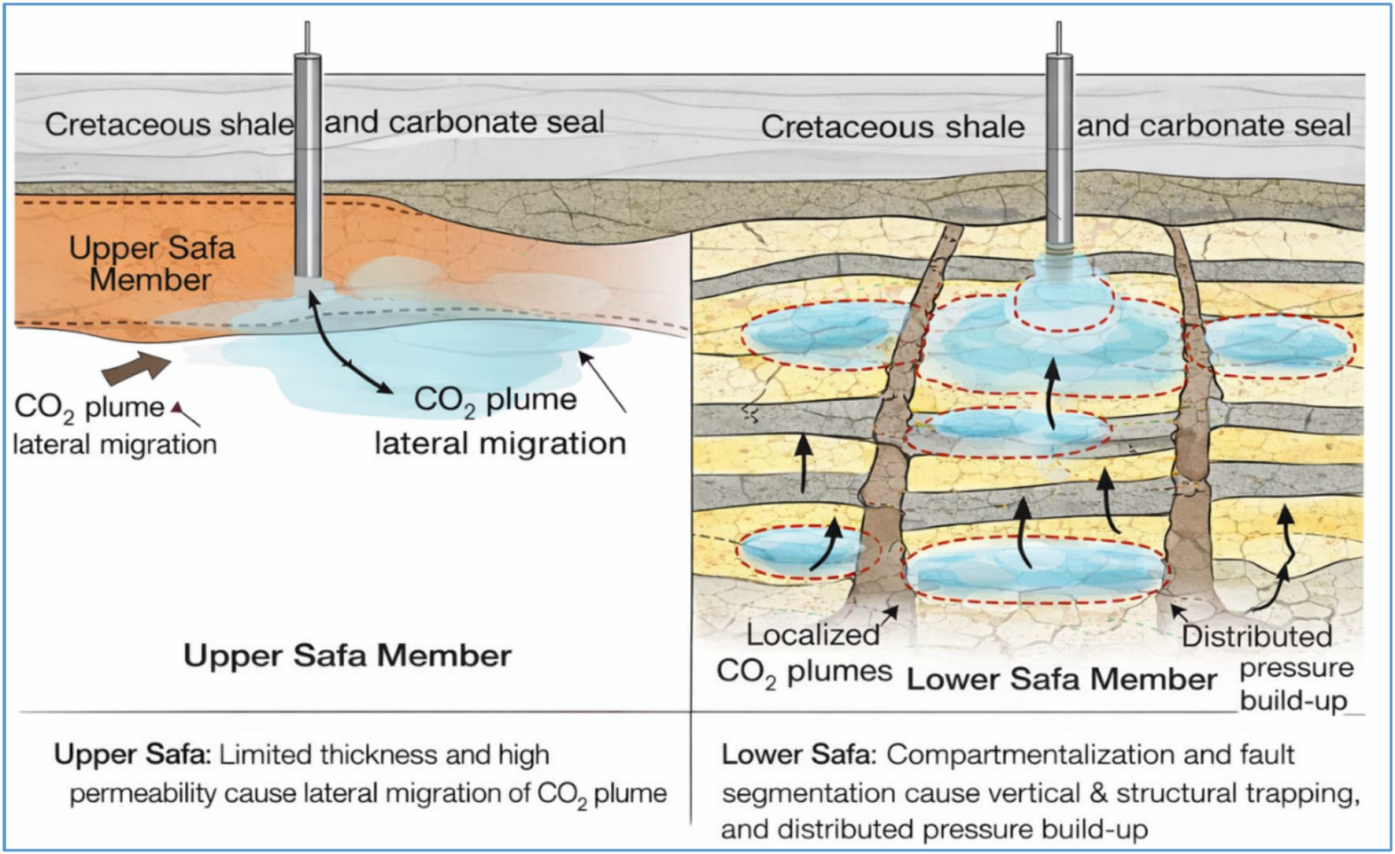
Conceptual representation of expected CO_2_ distribution and flow behavior within the upper and lower Safa members of the Obaiyed Field. *Conceptual illustration (not based on dynamic reservoir simulation).

The conceptual schematic (Fig. 10) summarizes how petrophysical heterogeneity, fault-controlled compartmentalization, and sealing relationships may influence CO_2_ distribution and trapping within the Safa reservoir system. The figure represents an integrated geological interpretation and does not depict simulated flow behavior and is intended for qualitative interpretation of reservoir comportment.

#### Injectivity constraints and pressure-limited storage behavior

To evaluate CO_2_ injectivity and storage constraints, reservoir pressure data from wells OBA-2–3, OBA-D5A, and OBA-D13 were analyzed (Table 6). Measured formation pressures indicate values of approximately 4200 psi at ~ 3100 m, 5950 psi at ~ 3930 m, and 4400 psi at ~ 3165 m, corresponding to calculated pressure gradients ranging from ~ 1.3 to ~ 1.5 psi/m. These gradients are consistent with near-hydrostatic to moderately elevated pressure conditions and show measurable variation between wells at comparable depths. Such variations indicate limited hydraulic communication across the reservoir and provide direct evidence for pressure compartmentalization, consistent with the interpreted fault-controlled segmentation of the Obaiyed Field.

**Table 6:** Reservoir pressure characteristics and injectivity implications.

Well	Depth (m)	Pressure (psi)	Gradient (psi/m)	Interpretation
OBA-2–3	~ 3100	~ 4205	~ 1.33	near-hydrostatic
OBA-D5A	~ 3930	~ 5947	~ 1.51	slightly elevated pressure
OBA-D13	~ 3165	~ 4408	~ 1.39	moderate pressure

From an injectivity perspective, these pressure conditions suggest that CO_2_ storage within the Lower Safa Member is primarily constrained by pressure buildup rather than by available pore volume. In tight to moderately permeable reservoirs, injection performance is controlled by the ability to maintain bottom-hole pressures below fracture and caprock integrity limits. The observed pressure regime therefore defines a finite operational window for safe CO_2_ injection. The influence of reservoir heterogeneity further reinforces this behavior. **Moreover,** core-derived permeability data indicate significant spatial variability, implying that pressure dissipation is likely to be non-uniform and controlled by localized flow pathways within fault-bounded compartments. As a result, injection-induced pressure buildup may be concentrated within individual compartments, limiting effective injectivity at the field scale.

Although dynamic reservoir simulation was not performed in this study, the integration of measured pressure data, core-derived petrophysical properties, and structural interpretation provides a consistent framework for evaluating pressure-limited storage behavior at the screening level. These results highlight that CO_2_ storage in the Lower Safa reservoir is governed by pressure constraints and compartment-scale flow behavior, which must be considered in future injection design and site-specific dynamic modeling.

### Structural framework and reservoir compartmentalization

**Fig. 11:**
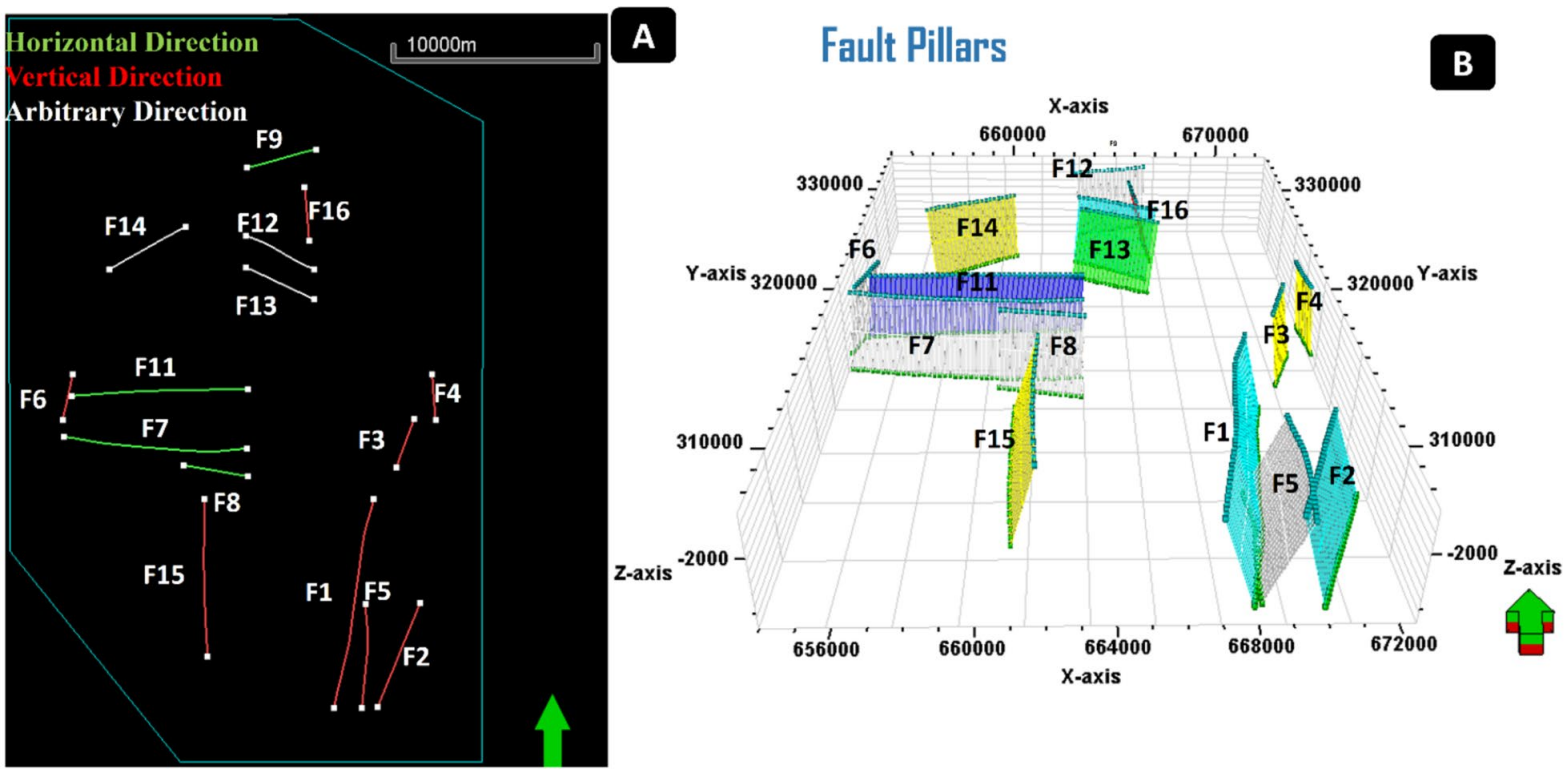
Quality control of the interpreted fault network for the three-dimensional structural model, illustrating (**A**) fault pillars in 2D seismic sections and (**B**) their spatial representation in the 3D model.

Quality control of the fault interpretation confirms that the interpreted fault network is internally well-defined and provides a geologically coherent structural basis for the three-dimensional model. Fault pillar analysis in both two- and three-dimensional views shows well-defined fault geometries and continuity, indicating reliable seismic picking and structural correlation (Fig. 11). The interpreted fault network is dominated by north–south–trending faults, with a subordinate set of west–east–trending structures, reflecting the inherited tectonic fabric of the Shushan Basin. The structural configuration defines the main structural compartments and pressure segmentation and provides the framework for subsequent facies and petrophysical modeling.

**Fig. 12:**
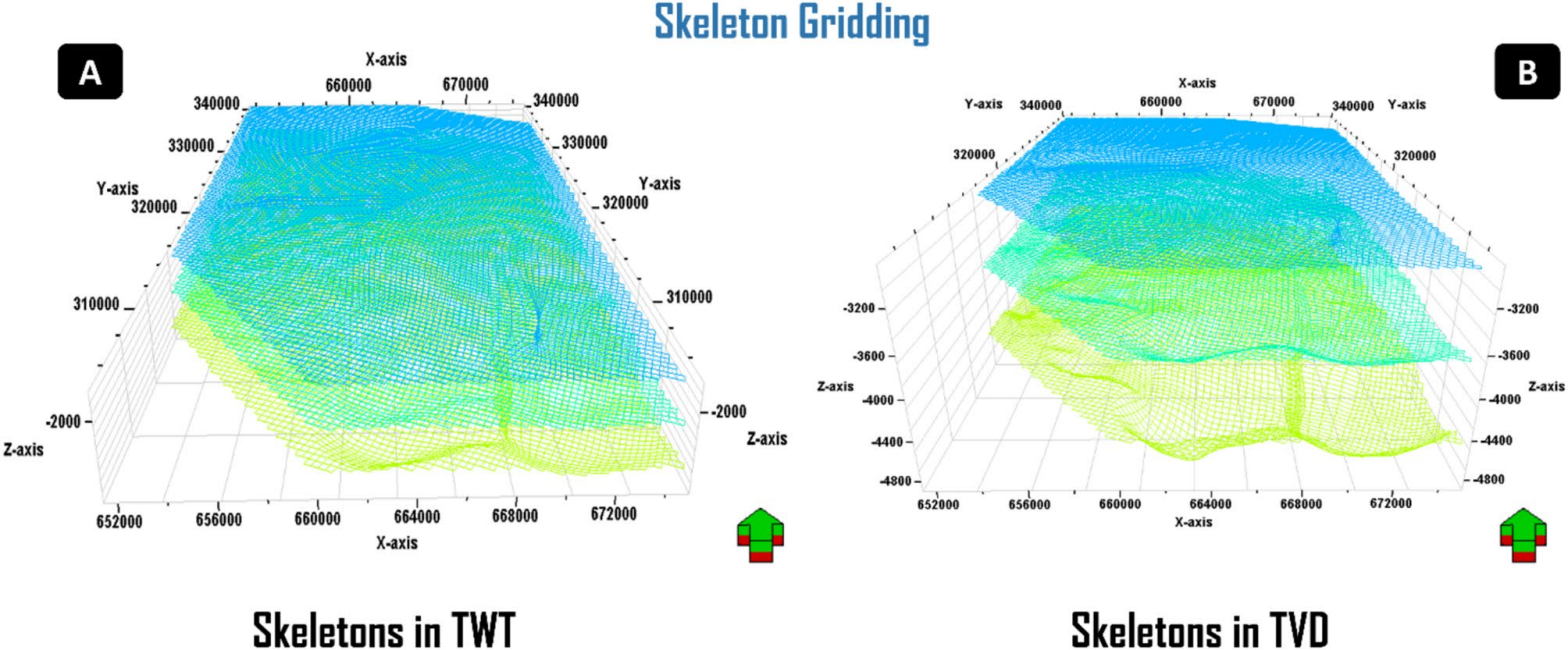
Skeleton grid of the structural framework constructed in (**A**) two-way travel time (TWT) and (**B**) true vertical depth (TVD) domains, illustrating the integration of interpreted faults and horizons used to define reservoir geometry and compartmentalization.

Following validation of the fault framework, a skeleton grid was constructed in both two-way travel time (TWT) and true vertical depth (TVD) domains to define the structural foundation of the three-dimensional geological model (Fig. 12). The integration of faults and horizons within the dual-domain grid preserves the interpreted structural geometry between the time and depth domains. This framework also provides the structural basis for subsequent facies and petrophysical modeling.

**Fig. 13:**
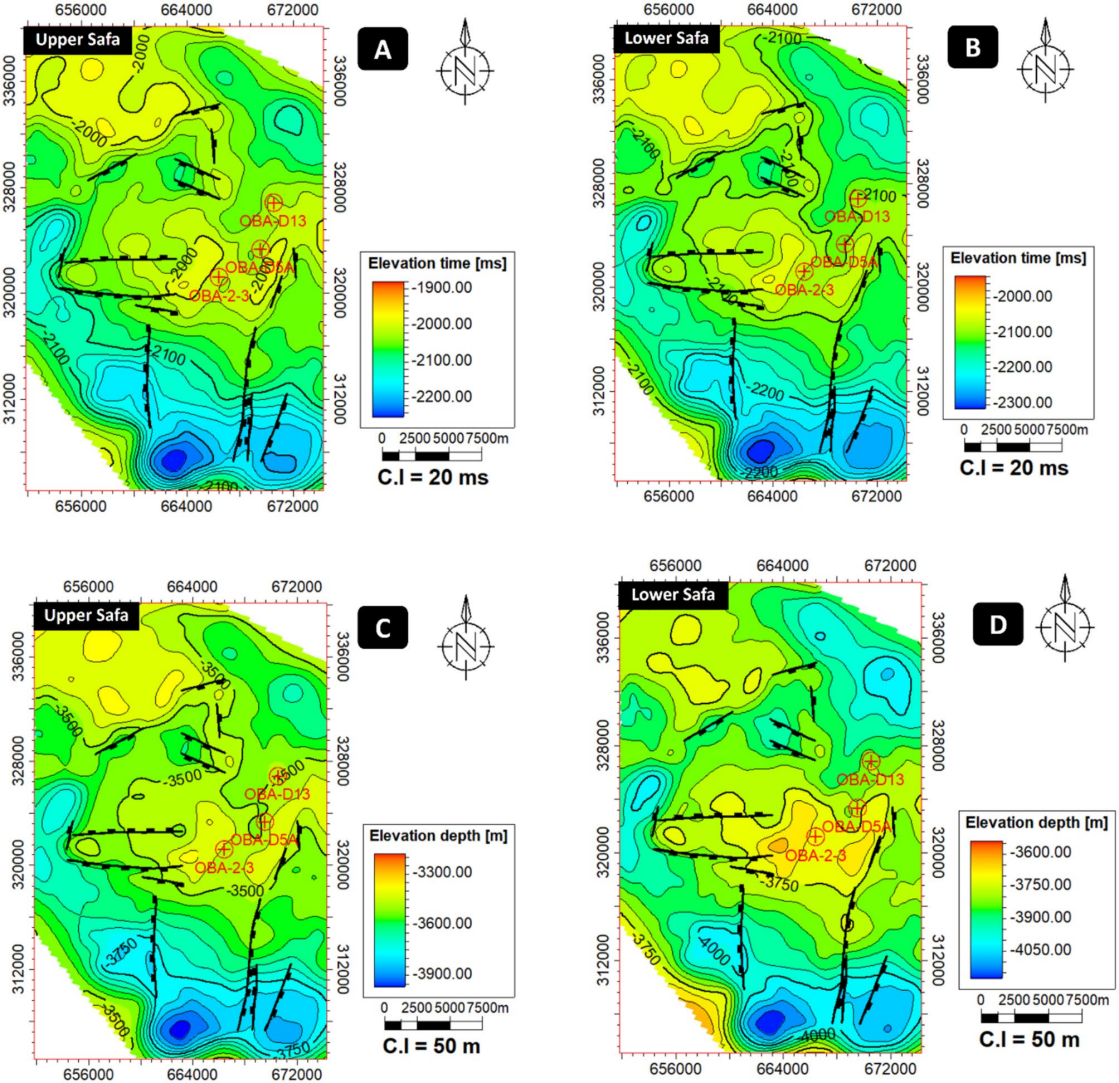
Two-way travel time (TWT) maps of the (**A**) Lower Safa and (**B**) Upper Safa members, and corresponding true vertical depth (TVD) structural maps of the (**C**) Upper Safa and (**D**) Lower Safa members, illustrating fault-controlled structural geometry and reservoir compartmentalization.

Two-way travel time (TWT) maps of the Lower Safa (Fig. 13A) and Upper Safa (Fig. 13B) members delineate reservoir extent and reveal significant structural variability associated with fault displacement across the study area. The corresponding depth-structure maps (Fig. 13C, D) define distinct structural closures and fault-bounded compartments, with the Lower Safa interval exhibiting greater structural relief and segmentation.

**Fig. 14:**
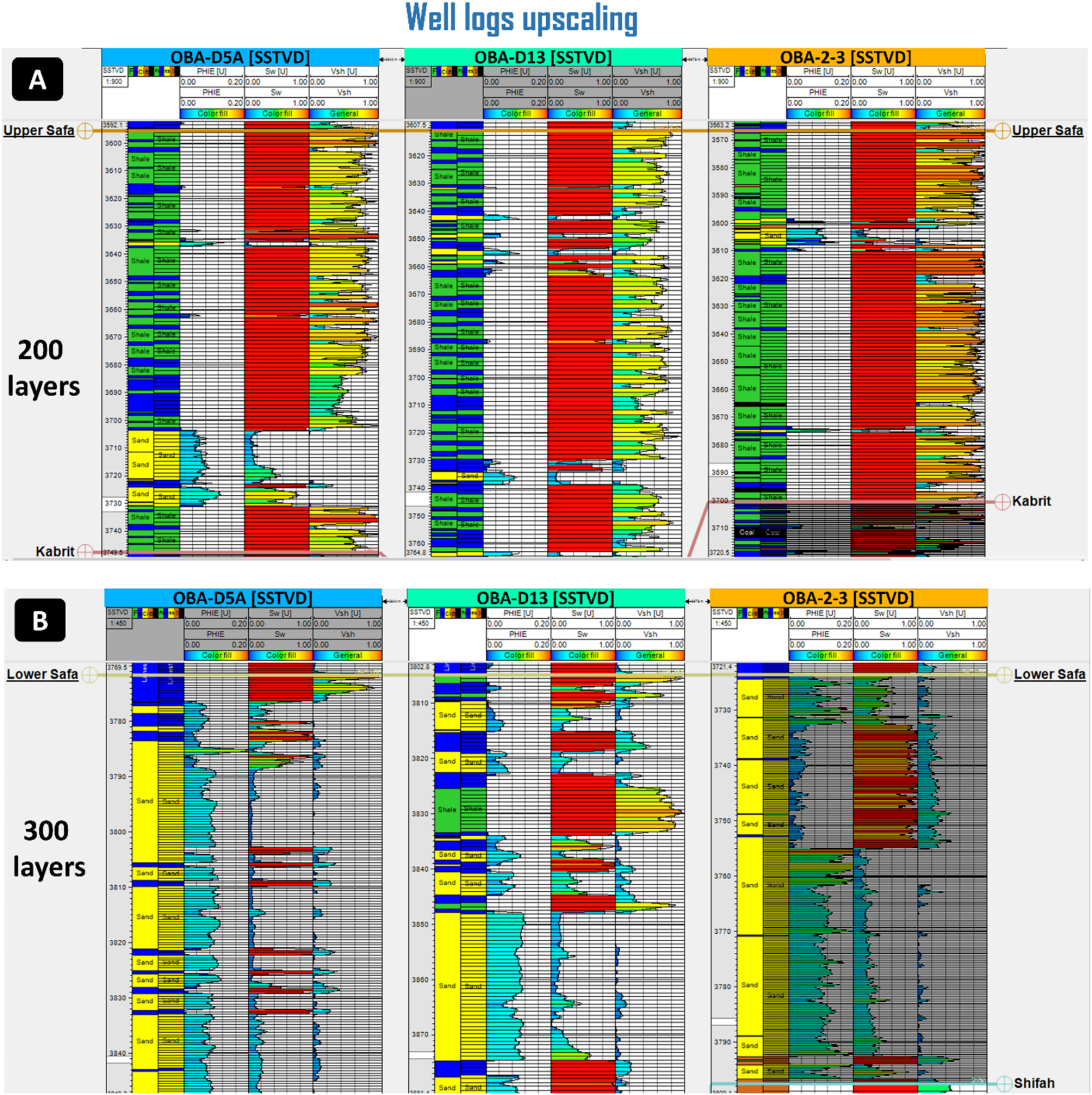
presents the upscaling of high-resolution wireline log data to the three-dimensional geological grid for the (**A**) Upper Safa Member (200 layers) and (**B**) Lower Safa Member (300 layers).

The upscaled well-log data used for three-dimensional geological modeling are illustrated in (Fig. 14).

### Static geological modelling

#### The 3D structural model

**Fig. 15:**
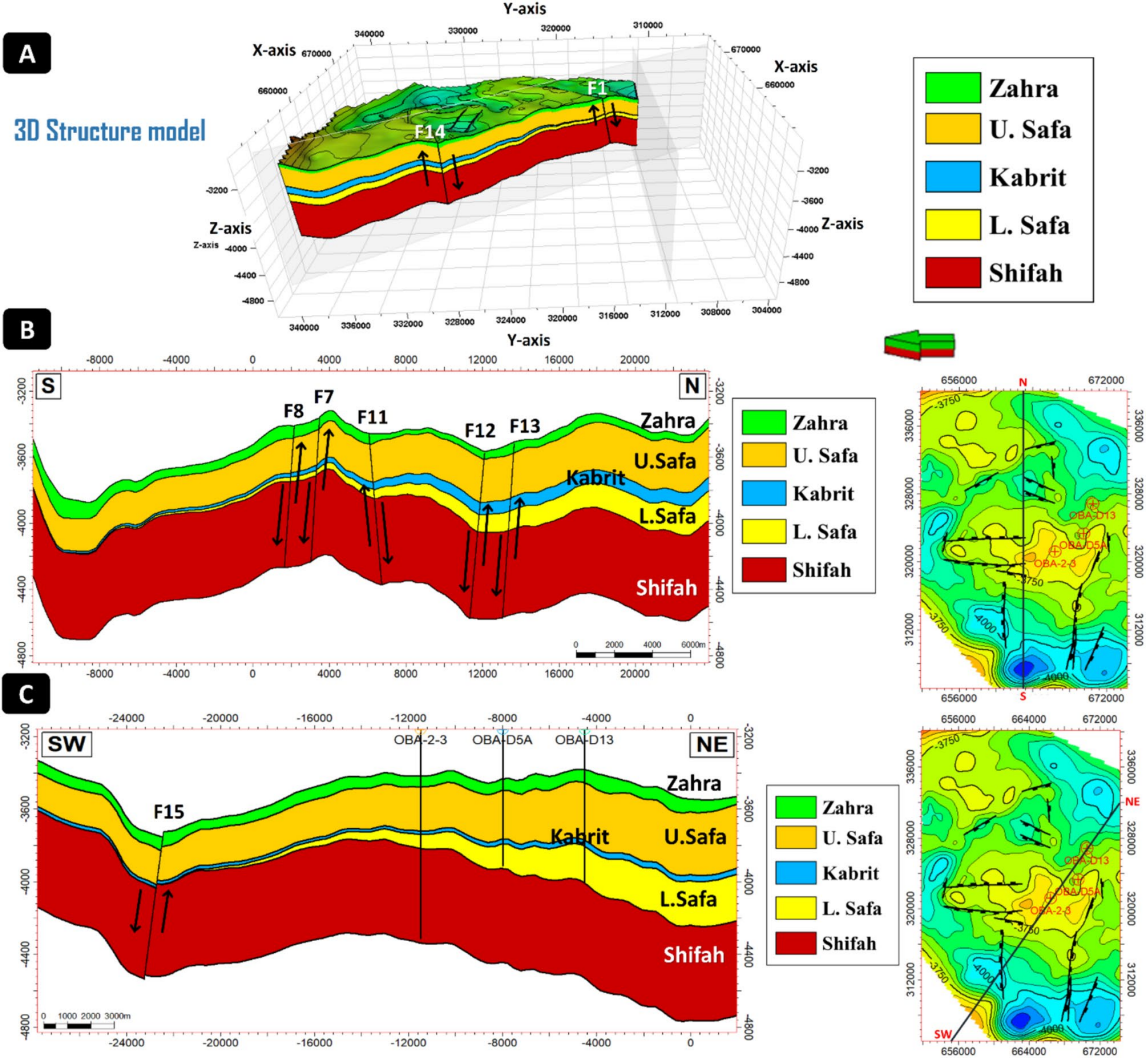
Three-dimensional structural model of the Khatatba Formation members in the Obaiyed Field. (**A**) Perspective view of the 3D structural framework showing interpreted horizons and fault architecture; (**B**) and (**C**) representative cross sections illustrating fault-bounded compartments and depth variations across the Jurassic succession within the Shushan Basin.

The 3D structural model integrates the interpreted seismic horizons, fault framework, lithology, and petrophysical properties to define reservoir geometry and structural configuration across the study area. Interpreted fault systems and stratigraphic horizons constrain the Jurassic succession and delineate fault-bounded compartments and depth variations within the Khatatba Formation (Fig. 15A–C).

#### Property modeling

**Fig. 16:**
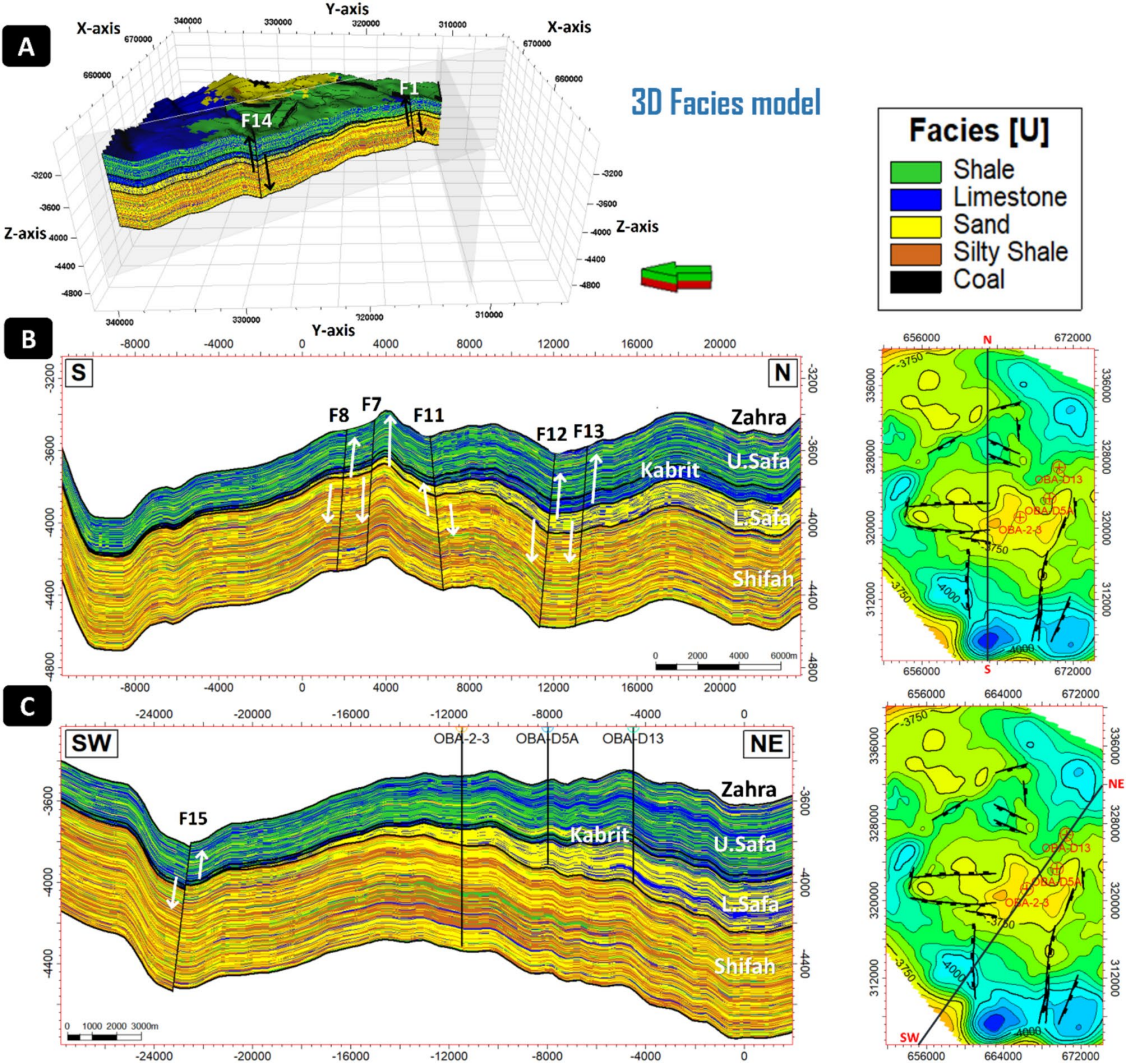
Three-dimensional facies models of the Middle Jurassic Khatatba Formation, Obaiyed Field. (**A**–**C**) Facies model showing the spatial distribution of reservoir and non-reservoir units with representative cross sections.

**Fig. 17:**
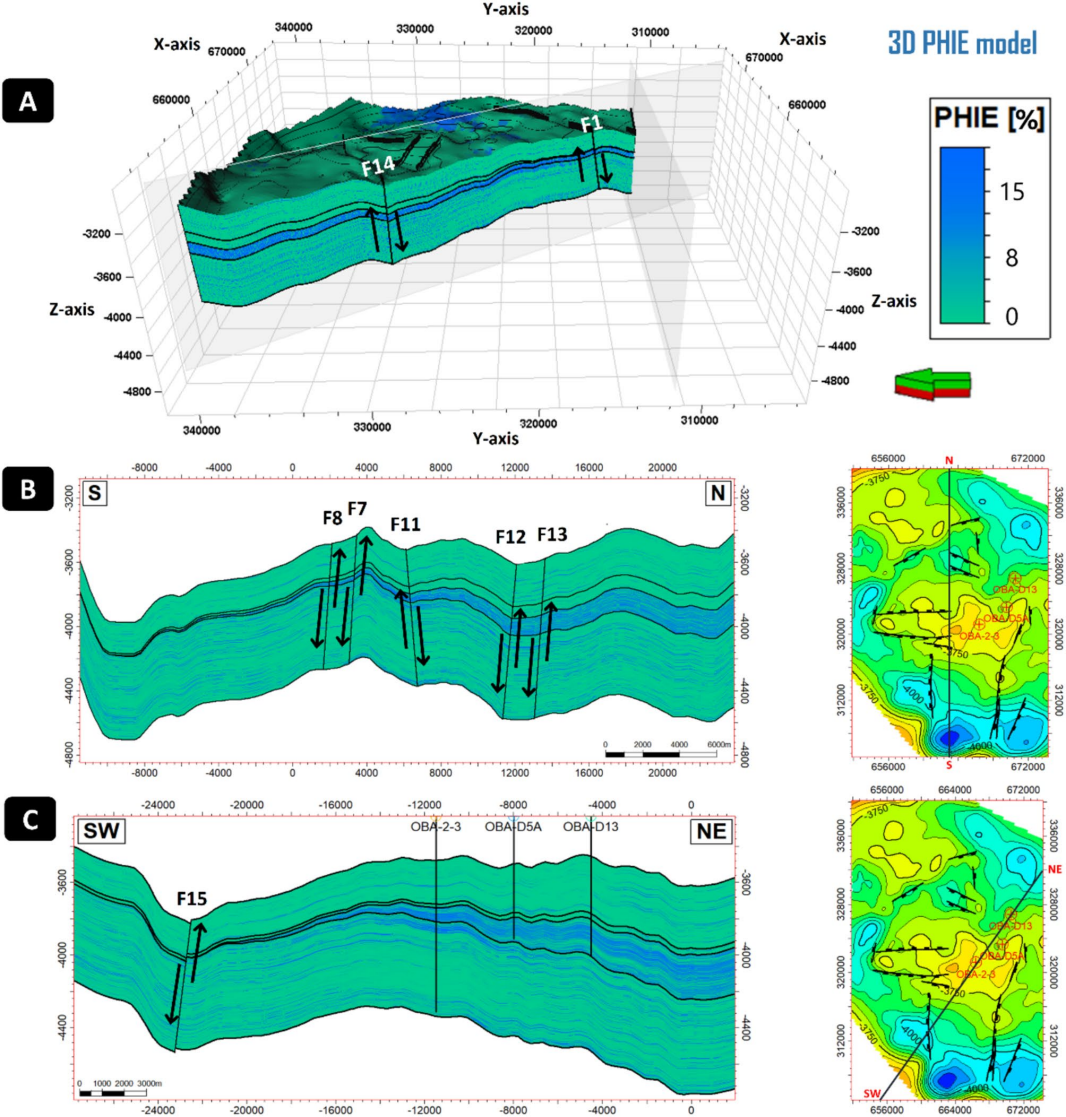
Three-dimensional petrophysical property model of the Middle Jurassic Khatatba Formation, Obaiyed Field. (**A**–**C**) effective porosity model illustrating lateral and vertical heterogeneity within reservoir compartments.

**Fig. 18:**
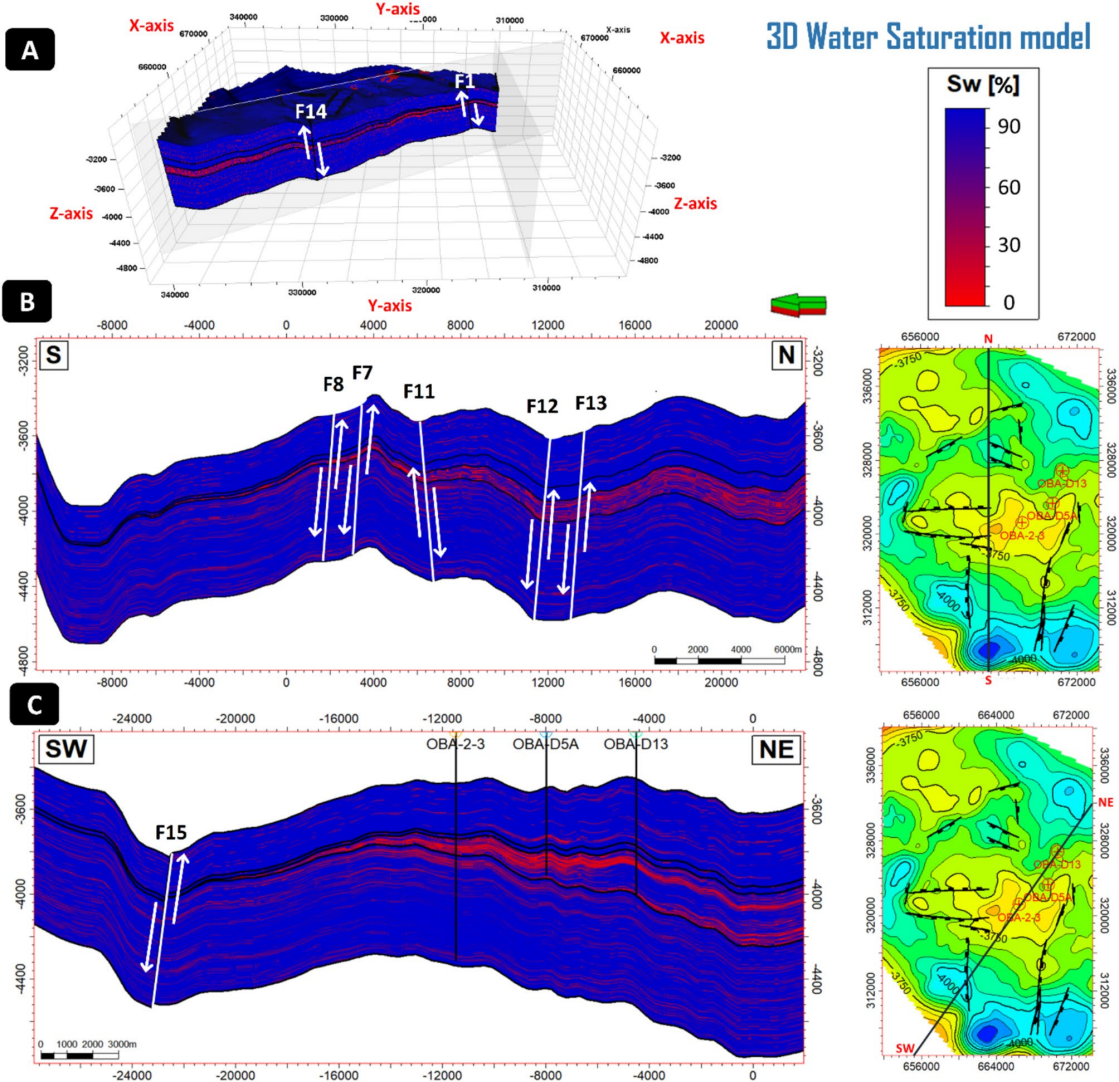
Three-dimensional saturation model of the Middle Jurassic Khatatba Formation, Obaiyed Field. (**A**–**C**) Water saturation distribution highlighting fluid distribution controlled by facies architecture and structural segmentation. Red colors indicate oil-saturated zones, whereas blue colors represent higher water saturation.

The 3D facies and property models depict the spatial distribution of reservoir characteristics within the Middle Jurassic Khatatba Formation (Figs. 16A–C, 17A–C, and 18A–C). Facies modeling highlights pronounced lateral and vertical heterogeneity associated with depositional architecture and fault-controlled compartmentalization, whereas **effective porosity** and **oil saturation** models reveal strong variability linked to facies distribution, structural segmentation, and reservoir connectivity. The integrated models reproduce the spatial organization of facies and petrophysical properties observed in the reservoir.

#### Model validation

**Fig. 19:**
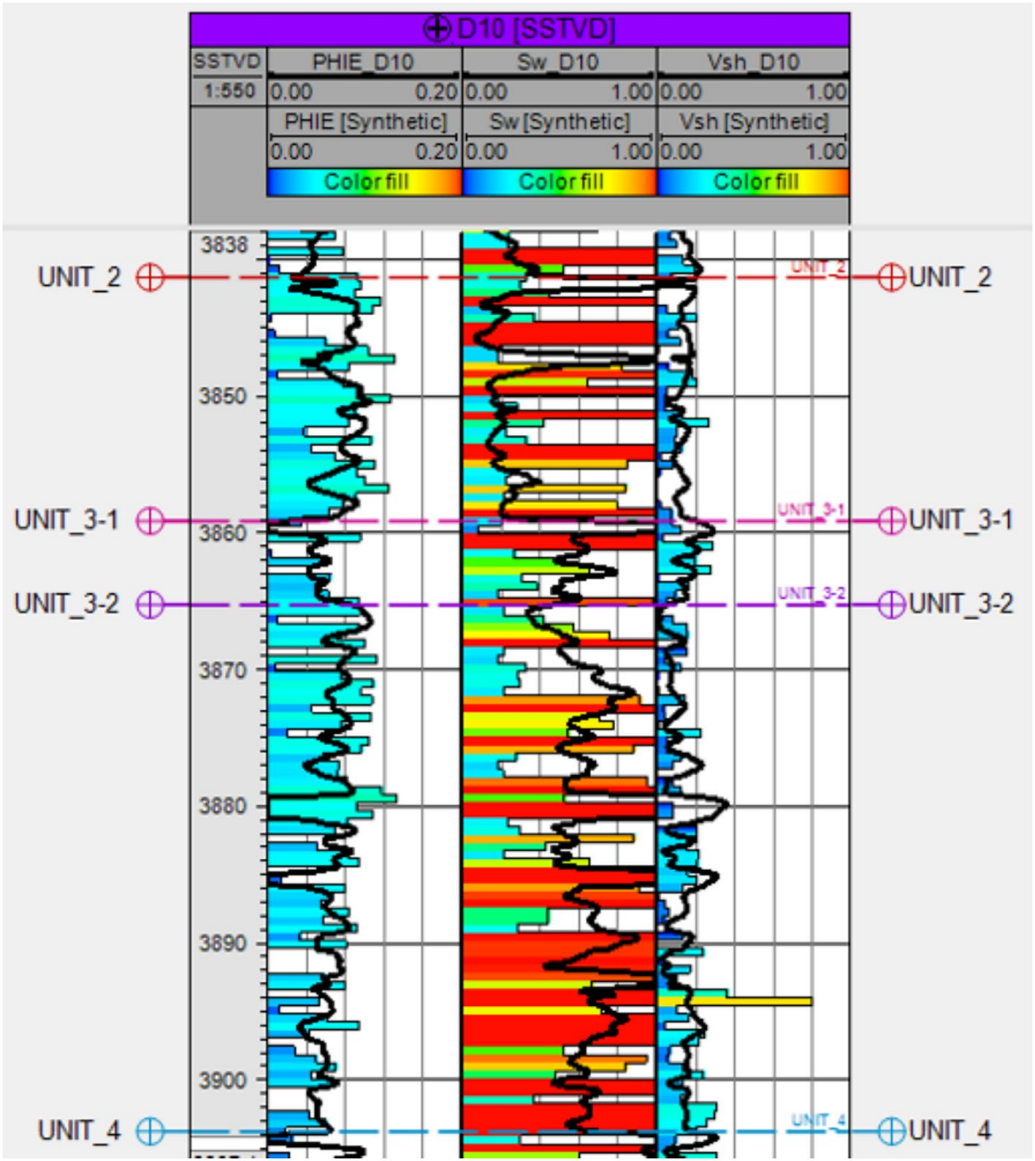
Validation of the petrophysical model at the D10 well. Bold curves represent the original measured wireline logs, while the color-filled tracks correspond to synthetic logs extracted from the 3D geological model at the X–Y location of the **D10 well**, illustrating the level of agreement between modeled and observed log responses.

The D10 well was used as an independent validation well to assess the performance of the petrophysical model. In the validation section (Fig. 19), bold curves represent measured well logs, while color-filled tracks show synthetic logs generated at the X–Y location of the D10 well. Quantitative comparison indicates strong agreement between measured and modeled effective porosity (PHIE), water saturation (Sw), and shale volume (Vsh). High correlation coefficients and low root mean square error (RMSE) values confirm that the model accurately reproduces porosity distribution, fluid-saturation behavior, and lithological variability (Table 7).

**Table 7:** Quantitative comparison between measured and synthetic petrophysical logs at the D10 validation well.

Petrophysical property	Correlation coefficient (R)	RMSE	Units
PHIE (effective porosity)	0.90	0.03	v/v
Sw (water saturation)	0.88	0.05	v/v
Vsh (shale volume)	0.92	0.04	v/v

**Fig. 20:**
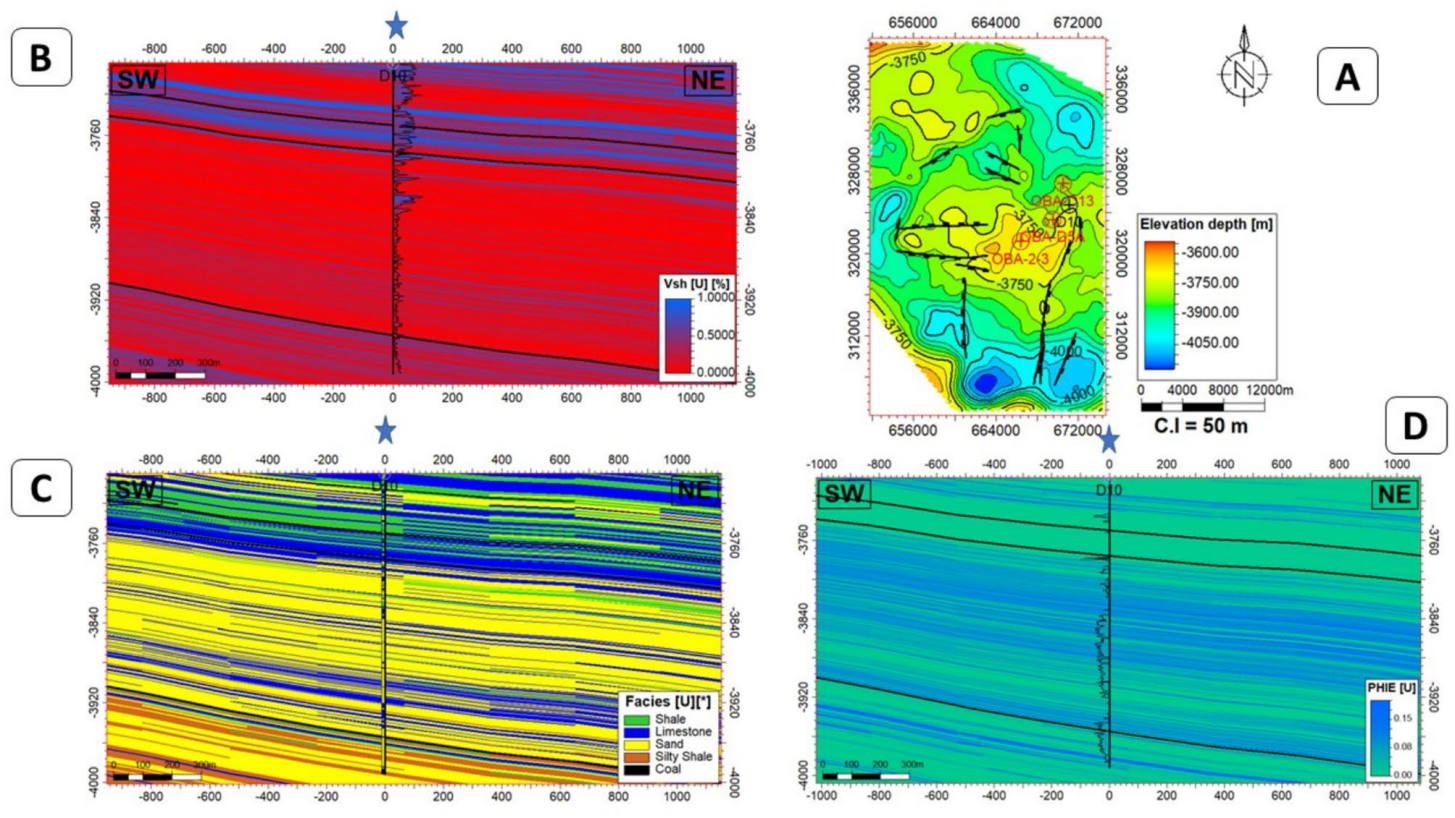
Model validation using the blind well (D10) within the 3D geological model. (**A**) Structural depth map showing the location of the blind well (D10) and the extracted validation section. (**B**) Cross-section of shale volume (Vsh) extracted from the 3D model along the blind well trajectory. (**C**) Corresponding facies model showing the predicted spatial distribution of lithofacies. (**D**) Effective porosity (PHIE) section extracted along the same profile.

Moreover, to evaluate the predictive performance of the 3D geological model, the independent blind well (D10) that was excluded from model construction was used for validation (Fig. 20). Cross-sections extracted through the blind well show a realistic spatial distribution of shale volume (Vsh), lithofacies, and effective porosity (PHIE), with stratigraphic boundaries and reservoir intervals accurately reproduced by the model. The comparison demonstrates the capability of the geological model to reproduce the subsurface architecture and petrophysical trends at an independent well location that was excluded from model construction. The close agreement between the predicted geological features and the blind-well log responses indicates that the model reliably reproduces the observed spatial heterogeneity.

#### Spatial distribution of reservoir properties

**Fig. 21:**
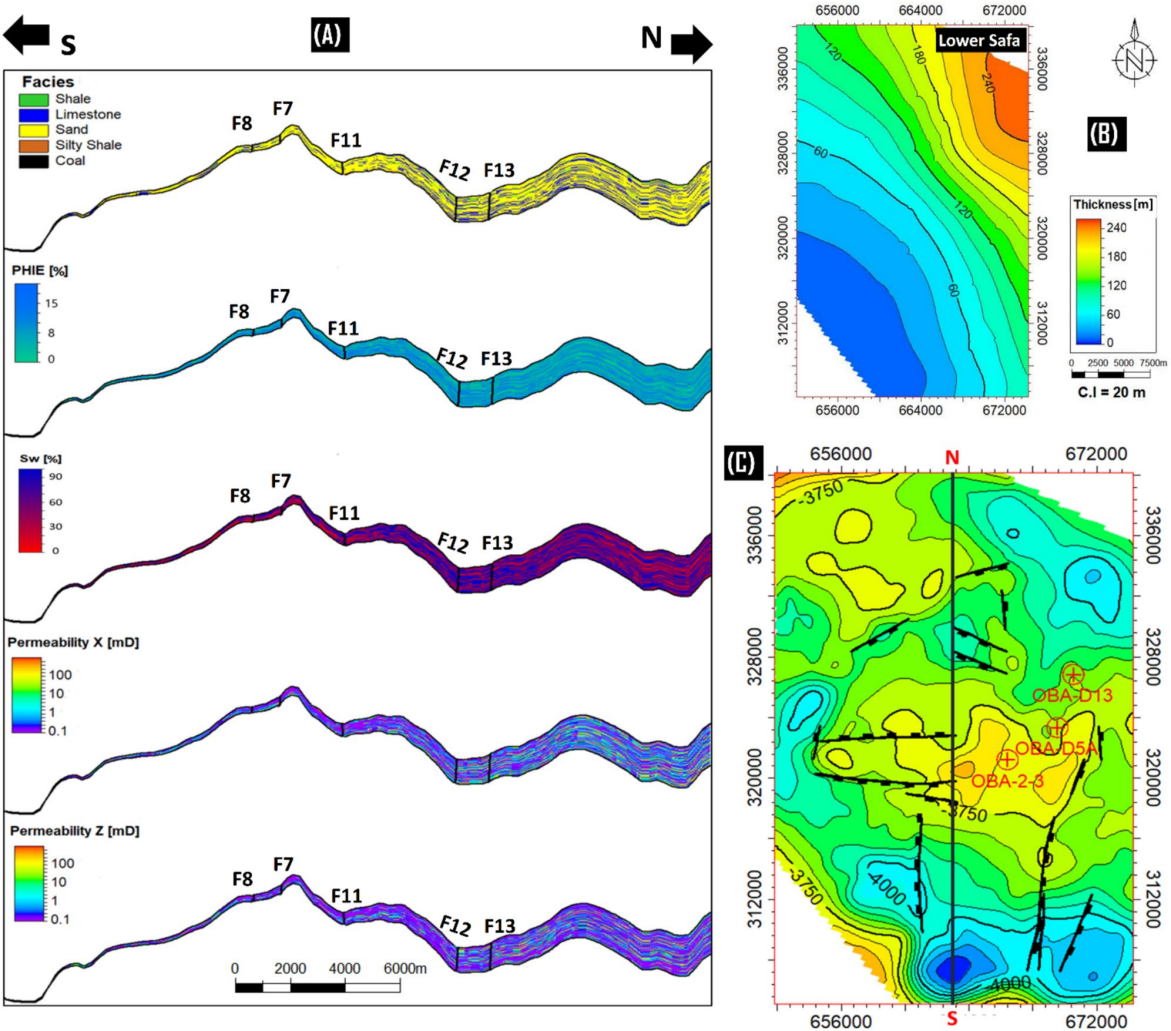
Representative cross-sections through the three-dimensional structural and petrophysical models. (**A**) Cross-sections through the three-dimensional structural and petrophysical models showing, from left to right, facies distribution, effective porosity (PHIE), water saturation (Sw), horizontal permeability (Kx), and vertical permeability (Kz). (**B**) Thickness map of the Lower Safa Member, illustrating a progressive increase in thickness toward the northeastern (NE) direction. (**C**) Location map showing the distribution of wells and the major fault systems intersecting the study area.

Cross-sections through the 3D model (Fig. 21) illustrate the lateral and vertical variability of geological and petrophysical properties and provide an integrated view of subsurface heterogeneity and fluid distribution. Facies and zonation patterns show clear stratigraphic continuity and lateral transitions controlled by structural configuration. Variations in shale volume (Vsh) reflect depositional and lithological heterogeneity, while effective porosity (PHIE) displays systematic spatial trends, with higher values associated with cleaner facies and structurally favorable zones. Corresponding water saturation (Sw) patterns, characterized by lower values within higher-porosity intervals, correspond to intervals characterized by lower water saturation. The extracted cross-sections reproduce the expected spatial relationships between facies architecture and petrophysical properties throughout the modeled reservoir.

### Fault seal analysis of the lower Safa member

**Fig. 22:**
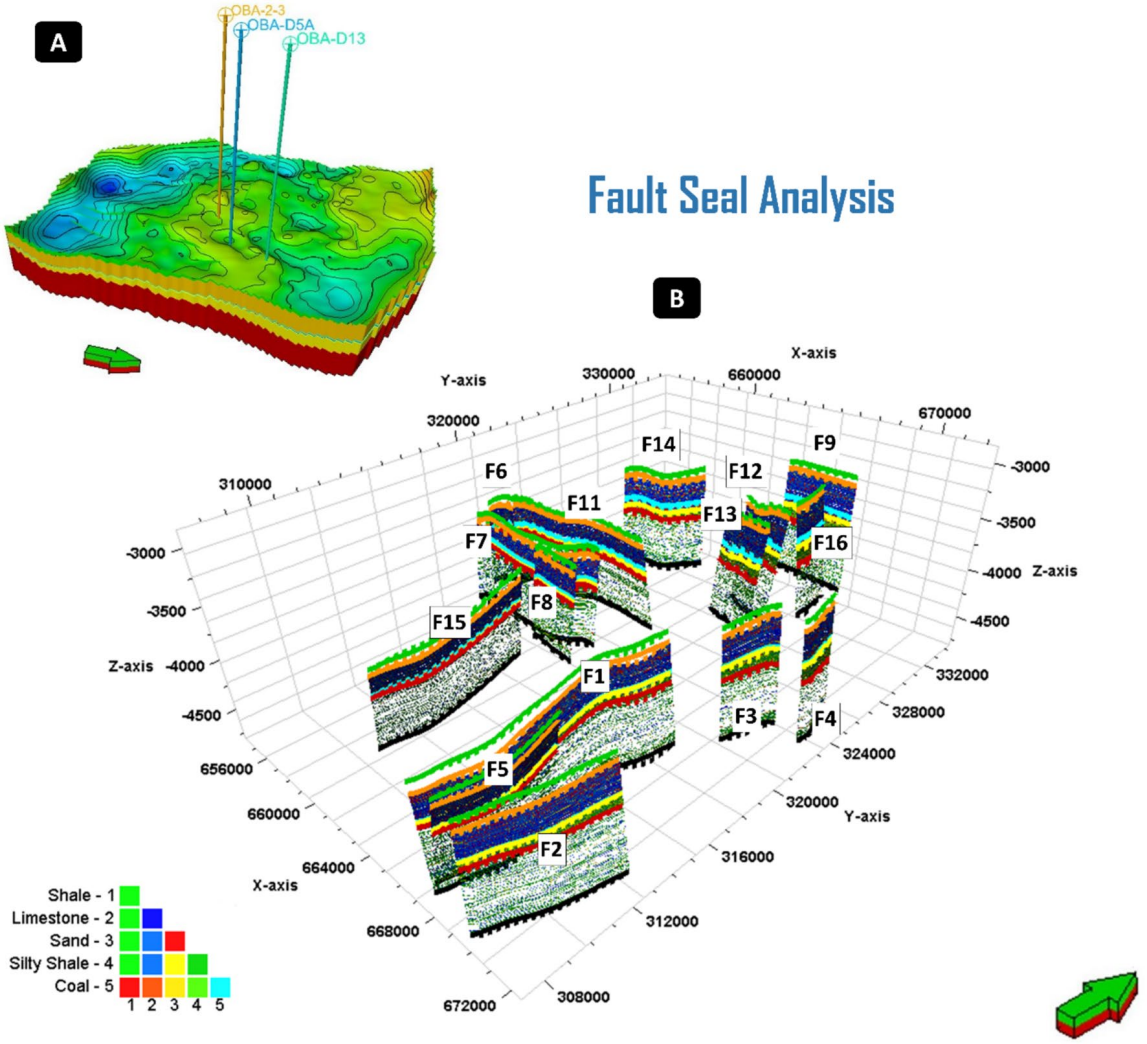
Fault seal analysis of the Lower Safa Member, Obaiyed Field. (**A**) Three-dimensional structural model showing the spatial distribution of the Lower Safa reservoir and the locations of the studied wells (OBA-2–3, OBA-D5A, and OBA-D13). (**B**) Fault juxtaposition panels for the interpreted fault network (F1–F16), illustrating lithological contacts across fault planes based on the 3D geological model.

**Fig. 23:**
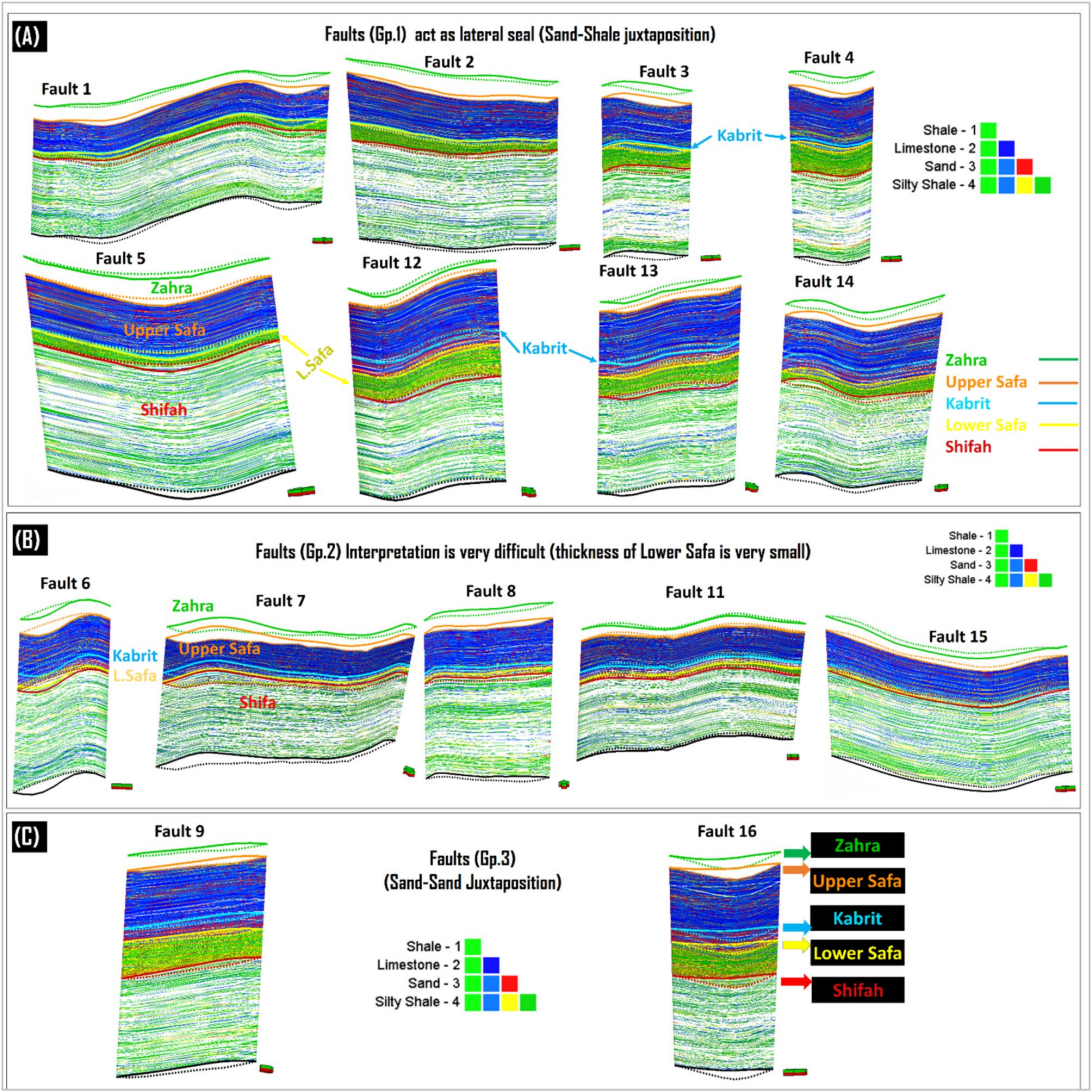
Fault seal analysis and lithological juxtaposition across the Lower Safa interval, Obaiyed Field. (**A**) Group-1 faults characterized by dominant sand–shale juxtaposition, (**B**) Group-2 faults have uncertain Interpretation, (**C**) Group-3 faults characterized by dominant sand–sand juxtaposition.

Fault-seal analysis of the Obaiyed Field reveals systematic variations in fault behavior that exert a first-order control on lateral fluid containment within the Lower Safa Member. The analysis is based on fault juxtaposition relationships extracted from the three-dimensional geological model, where sand–shale contacts are interpreted as sealing or baffle-prone configurations, whereas sand–sand contacts indicate potentially transmissive pathways. The fault seal maps (Fig. 22) demonstrate spatial variability in sealing behavior across the interpreted fault network. Three principal fault groups were identified based on lithological juxtaposition: (i) faults dominated by sand–shale contacts (Fig. 23A), (ii) faults where sealing behavior remains uncertain because of reservoir thinning and limited stratigraphic resolution (Fig. 23B), and (iii) faults characterized predominantly by sand–sand juxtaposition (Fig. 23C). This qualitative classification provides the structural framework for the subsequent quantitative fault seal evaluation.

#### Enhanced fault seal characterization

**Fig. 24:**
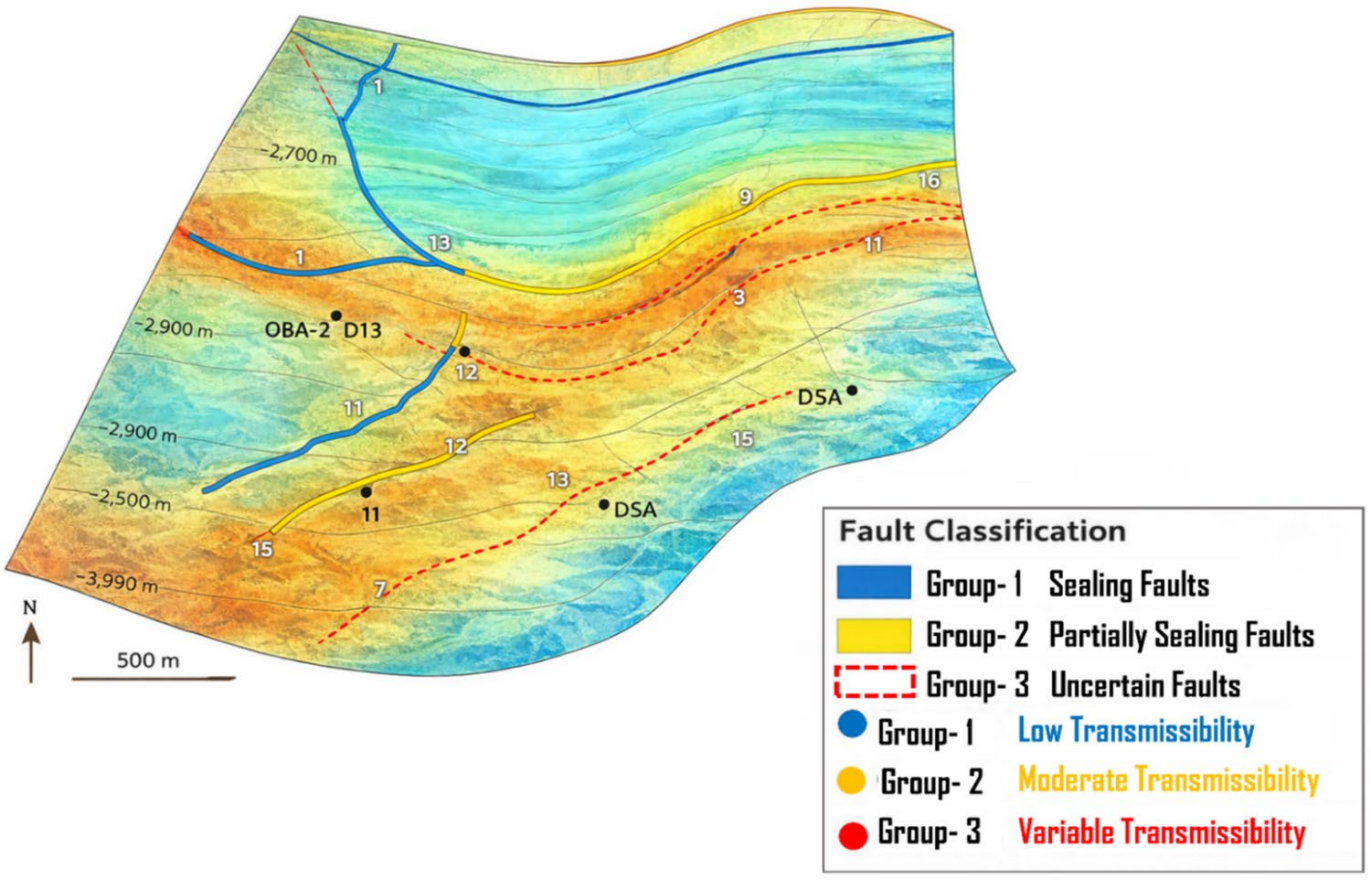
Integrated faults classification map showing structurally isolated compartments bounded by low-transmissibility sealing faults (Group 1), moderately connected compartments associated with partially sealing faults (Group 2), and zones where sealing behavior remains uncertain (Group 3).

The preliminary fault grouping established from lithological juxtaposition was subsequently evaluated using quantitative fault-seal parameters, including Fault Gouge Ratio (FGR), predicted fault permeability, transmissibility multipliers, and effective cross-fault transmissibility. Integration of these complementary indicators provides an independent assessment of sealing efficiency beyond simple stratigraphic juxtaposition. Faults 1, 12, 13, and 14 consistently exhibit high FGR values together with low fault permeability and transmissibility, supporting their classification as effective sealing faults. Faults 9 and 16 display intermediate values for these parameters, consistent with partially sealing (baffle) behavior, whereas faults 6, 7, 8, 11, and 15 remain difficult to classify because reduced reservoir thickness limits the reliability of quantitative fault-property estimates. The resulting integrated classification is summarized in (Table 8) and illustrated in (Fig. 24).

**Table 8:** Quantitative fault seal classification.

Group	Faults	FGR	Transmissibility	Fault Perm	Behavior	Hydraulic interpretation
Group 1	1, 12, 13, 14	High	Low	Low	Sealing	Strong containment
Group 2	9, 16	Moderate	Moderate	Moderate	Baffle	Limited pressure communication
Group 3	6, 7, 8, 11, 15	Variable	Uncertain	Variable	Uncertain	Injection risk zones

The final integrated fault classification map of the Lower Safa reservoir showing the spatial distribution of sealing, partially sealing, and uncertain faults derived from a multi-parameter fault seal analysis is shown in (Fig. 24).

#### Fault gouge ratio (FGR)

**Fig. 25:**
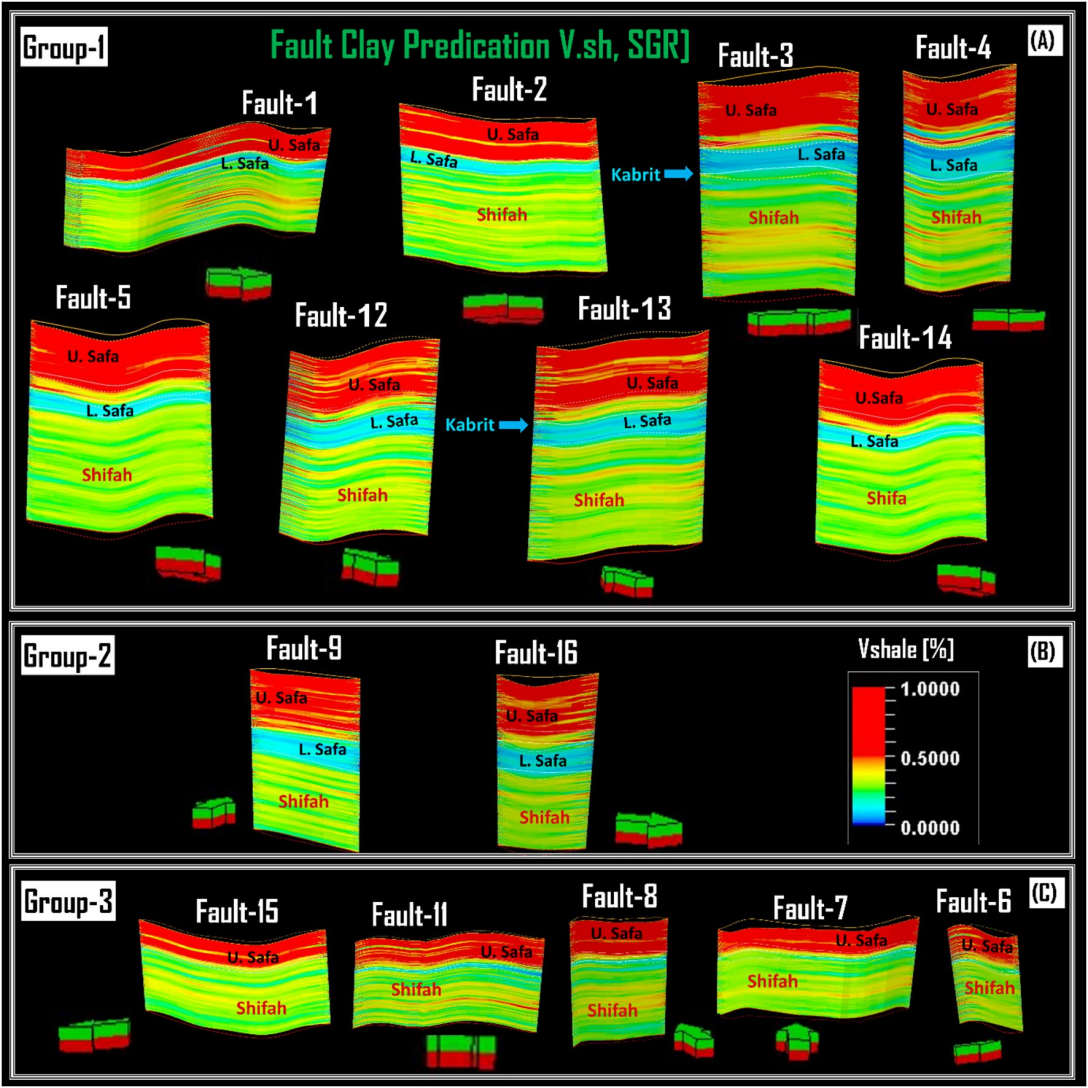
Fault Gouge Ratio (FGR) distribution for the mapped faults within the Lower Safa reservoir. Faults are grouped according to their interpreted sealing behavior derived from juxtaposition analysis. The spatial variability of FGR highlights differences in sealing efficiency among the mapped fault systems and provides a quantitative basis for subsequent fault permeability and transmissibility analyses.

Following the lithological juxtaposition assessment, **Fault Gouge Ratio (FGR)** was evaluated to quantify the potential development of clay-rich fault gouge within the mapped fault network (Fig. 25). The calculated FGR distributions reveal substantial variability among the interpreted faults, reflecting differences in fault displacement, stratigraphic architecture, and shale incorporation into the fault zone. Faults that previously identified as sealing through juxtaposition analysis generally exhibit elevated FGR values, indicating greater clay enrichment and enhanced sealing potential. In contrast, faults classified as partially sealing or uncertain display lower and more heterogeneous FGR distributions, suggesting reduced fault gouge development and a greater possibility of localized hydraulic communication. The observed variability in FGR supports the interpretation that fault sealing behavior within the Lower Safa reservoir cannot be explained only by lithological juxtaposition. Instead, sealing efficiency appears to be controlled by the combined effects of stratigraphic juxtaposition and fault-rock properties. The calculated FGR values provide an independent quantitative constraint on fault classification prior to permeability analysis.

#### Fault permeability

**Fig. 26:**
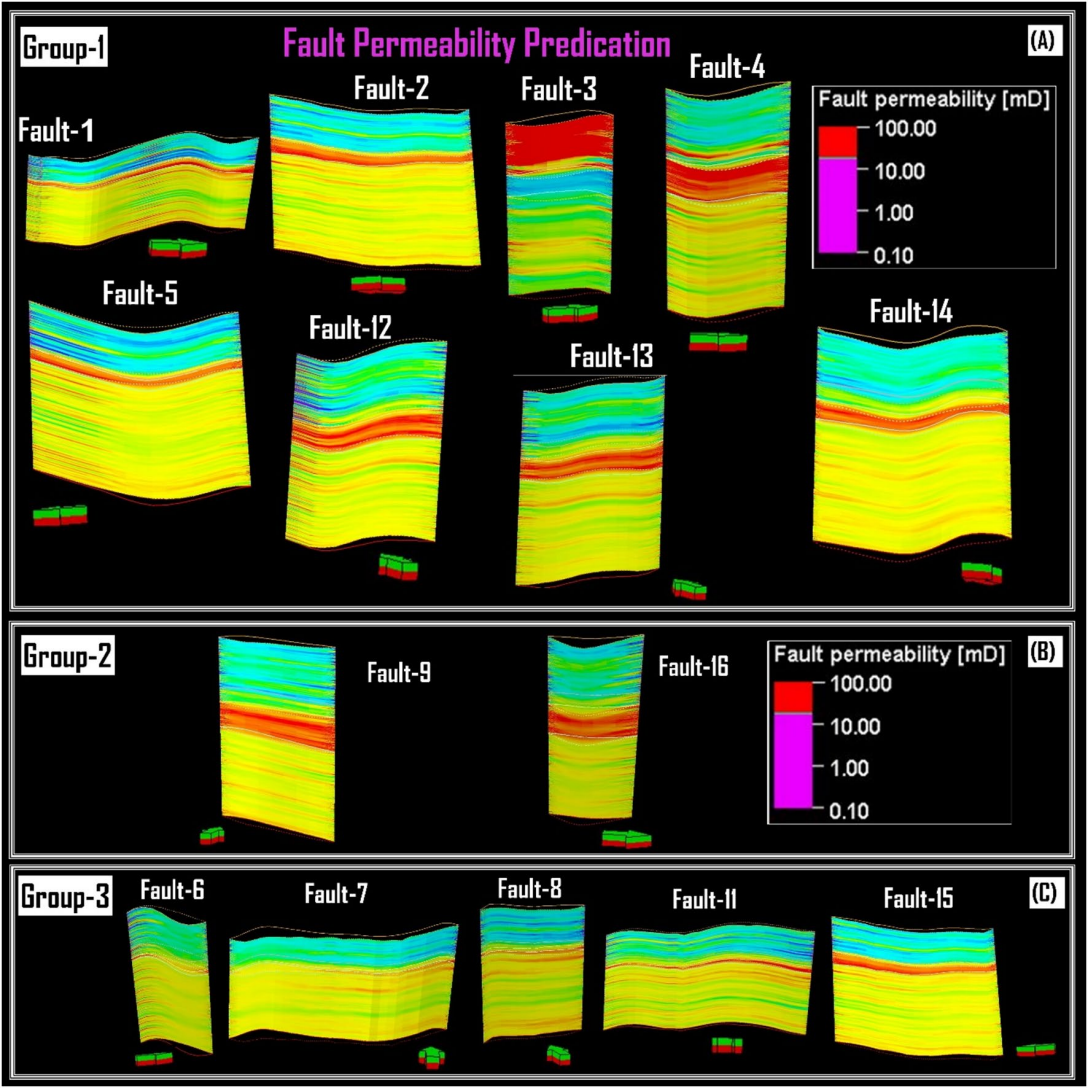
Predicted fault permeability distributions for the mapped faults grouped according to the integrated fault classification, illustrating spatial variations in fault hydraulic properties across the Lower Safa reservoir.

Predicted **fault permeability** distributions reveal substantial variability among the mapped faults (Fig. 26). Faults classified as sealing generally exhibit lower permeability values, consistent with enhanced fault-rock development and reduced hydraulic communication across fault planes. Partially sealing faults display intermediate permeability values, whereas faults assigned to the uncertain category exhibit greater spatial variability, suggesting heterogeneous fault-rock properties and locally variable sealing behavior. The observed permeability patterns are broadly consistent with the fault classification derived from lithological juxtaposition and fault gouge analyses, providing for the interpreted compartmentalization framework.

#### Fault transmissibility multiplier

**Fig. 27:**
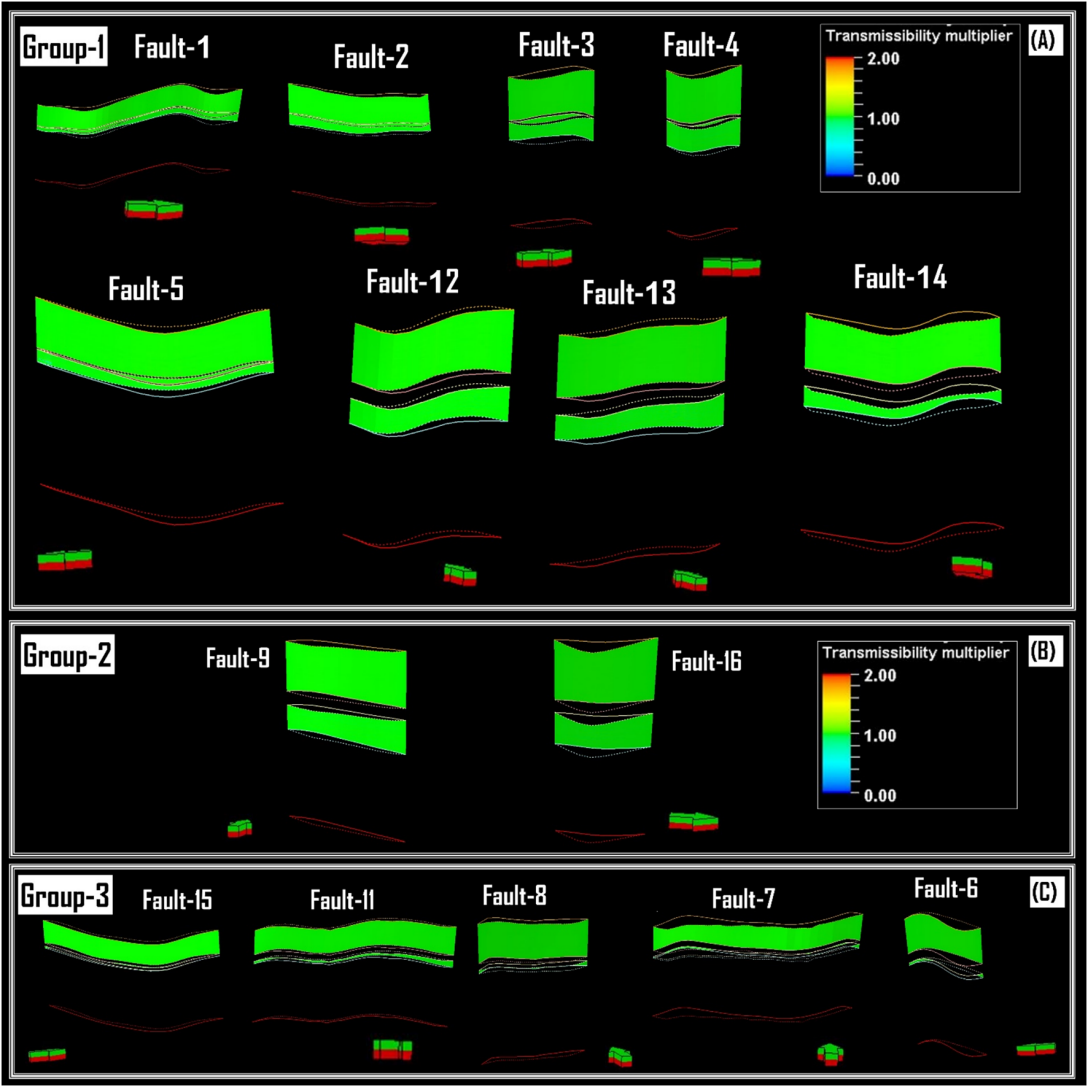
Quantitative assessment of fault-controlled flow behavior within the Lower Safa reservoir. Transmissibility multiplier distribution for all mapped faults, representing the relative reduction of cross-fault fluid flow compared to unfaulted reservoir conditions.

To quantitatively evaluate cross-fault fluid communication, transmissibility-based metrics were calculated for all interpreted faults within the Lower Safa reservoir. The transmissibility multiplier results (Fig. 27) indicate substantial reductions in cross-fault flow relative to unfaulted reservoir conditions, confirming that the fault network exerts a significant control on reservoir connectivity. Faults assigned to Group 1 exhibit the lowest transmissibility values and are therefore interpreted as effective hydraulic barriers capable of restricting lateral fluid migration. Group 2 faults display intermediate transmissibility values, suggesting partially sealing behavior that may permit limited pressure communication while still acting as flow baffles. In contrast, Group 3 faults show greater variability in transmissibility, reflecting uncertainty in sealing efficiency and the potential for localized fluid communication.

#### Effective cross-fault transmissibility

**Fig. 28:**
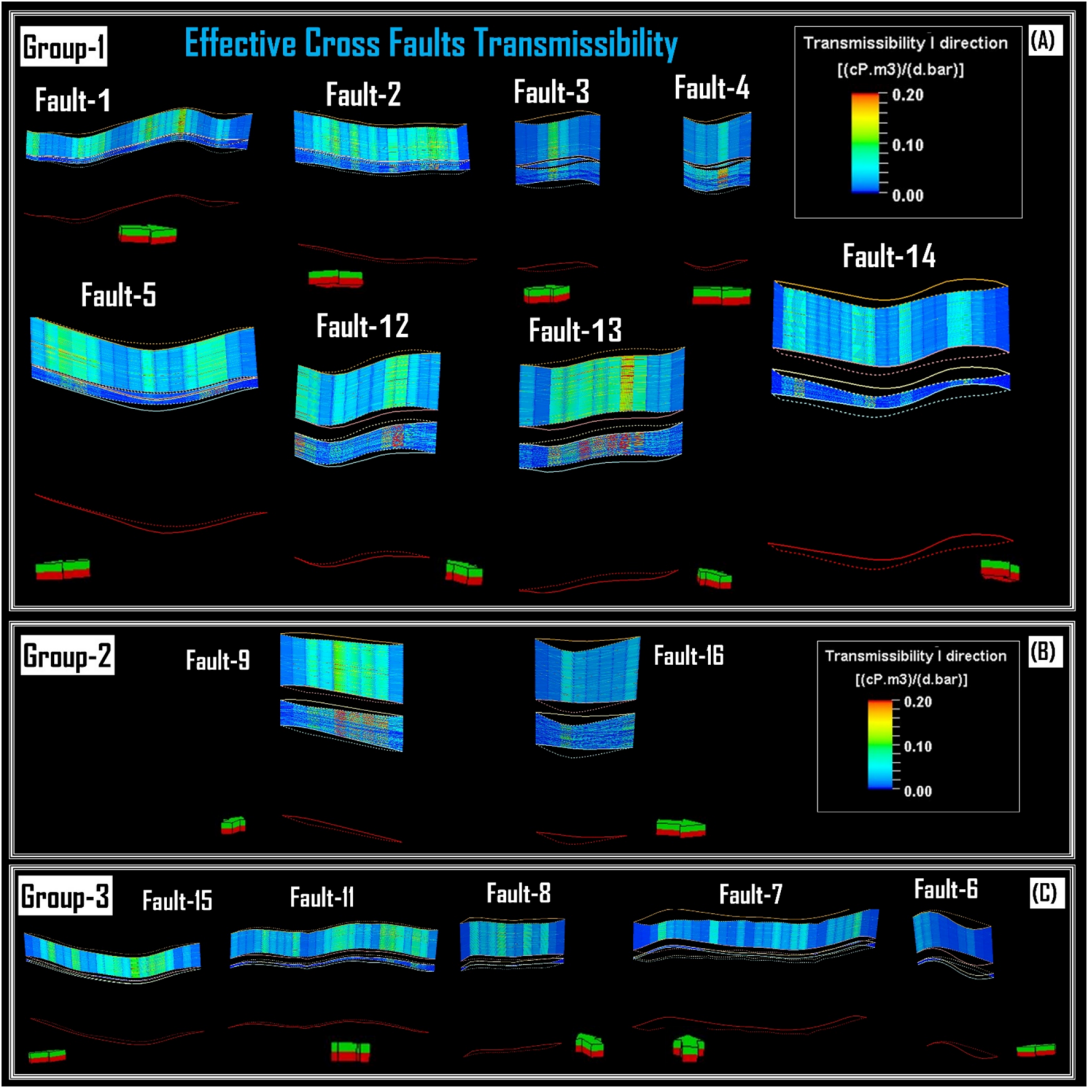
Quantitative assessment of fault-controlled flow behavior within the Lower Safa reservoir. Effective cross-fault transmissibility calculated from fault permeability, fault thickness, and reservoir properties, providing a quantitative measure of hydraulic communication across fault planes.

The effective cross-fault transmissibility analysis (Fig. 28) provides additional support for this classification. Low transmissibility values dominate across the Group 1 fault network, whereas Group 2 and Group 3 faults display progressively higher and more heterogeneous transmissibility distributions. The agreement between the transmissibility multiplier and effective transmissibility results demonstrates that the structural segmentation identified from juxtaposition analysis is reflected in the hydraulic behavior of the fault system.

Additional quantitative fault-rock property analyses, including fault Thickness, and Effective Cross Faults Permeability calculations, are provided in the Supplementary Information (Figs. S2–S3). These analyses support the fault classification framework and are reliable with the transmissibility-based interpretation presented in (Figs. 26, 27).

### CO_2_ Storage capacity results

CO_2_ storage capacity estimates derived from the three studied wells demonstrate pronounced vertical and lateral variability between the Upper and Lower Safa members (Tables 9, 10 and 11), consistent with the stratigraphic architecture, petrophysical heterogeneity, and fault-controlled compartmentalization described earlier. Across all wells, the Upper Safa Member contributes negligible storage capacity (MCO_2_ < 1 t), reflecting its limited effective pore volume, moderate porosity, and relatively high irreducible water saturation. In contrast, the Lower Safa Member accounts for nearly all of the estimated storage potential, with individual subunits yielding capacities that are one to three orders of magnitude higher.

**Table 9:** CO_2_ storage capacity calculation for the Upper and Lower Safa members in Well OBA-2–3.

Well: OBA 2–3		Upper safa	Lower Safa		
			Unit-1	Unit-4	Unit-5
Total Volume	Area (m^2^)	191340.95	261895955.4	261895955.4	261895955.4
	Thickness (m)	5.64	4.5	8	30.7
	Vt (m^3^)	1079162.958	1178531799	2095167643	8040205831
Vpv = PHIE* Vt		121945.4143	173244174.5	375035008.1	956784493.8
Space Volume (1-Swi)		0.72	0.61	0.44	0.73
Average effective porosity		0.113	0.147	0.179	0.119
MCO_2_ (Kg)		513.908462	618552.0834	965855.784	4088130.846
MCO_2_ (Ton)		0.513908462	618.5520834	965.855784	4088.130846
GIIP (m^3^)		22476978.76	27053810289	42243943315	178803886207

*Constant calculation parameters (ρCO_2_, B, and E) are identical for all wells.

**Table 10:** CO_2_ storage capacity calculation for the Upper and Lower Safa members in Well OBA-D-13.

OBA-D13 Well		Upper Safa	Lower Safa		
			Unit-1	Unit-2	Unit-3
					Unit-3–1
Total Volume	Area (m^2^)	191340.95	261895955.4	261895955.4	261,895,955.4
	Thickness (m)	6.5	3.5	16.154	8
	Vt (m^3^)	1243716.175	916635843.9	4230667263	2095167643
Vpv = PHIE* Vt		114421.8881	79747318.42	444220062.7	205326429
Space volume (1-Swi)		0.82	0.63	0.85	0.8
Average effective porosity		0.092	0.087	0.105	0.098
MCO_2_ (Kg)		549.1750033	294065.7446	2210064.221	961441.0039
MCO_2_ (Ton)		0.549175003	294.0657446	2210.064221	961.4410039
GIIP (m^3^)		24019442.75	12861647514	96662285634	42050852665

*Constant calculation parameters (ρCO_2_, B, and E) are identical for all wells.

**Table 11:** CO_2_ storage capacity calculation for the upper and lower Safa members in Well. OBA-D-5.

Well OBA- D5		Upper Safa	Unit-1	Unit-2	Unit-3		Unit-4	Unit-5
					Unit-3–1	Unit-3–2		
Total Volume	Area (m^2^)	191340.95	261895955.4	261895955.4	261895955.4	261895955.4	261895955.4	261895955
	Thickness (m)	8.7	2.4	11.6	18.14	18	3.5	1.5
	Vt (m^3^)	1664666.265	628550292.9	3037993083	4750792631	4714127197	916635843.9	392843933
Vpv = PHIE* Vt		144825.9651	70397632.81	270381384.3	456076092.6	386558430.2	79747318.42	35748797.9
Space volume (1-Swi)		0.79	0.67	0.93	0.915	0.82	0.59	0.65
Average effective porosity		0.087	0.112	0.089	0.096	0.082	0.087	0.091
MCO_2_ (Kg)		669.6707366	276070.9168	1471795.717	2442565.397	1855311.345	275394.9036	136007.419
MCO_2_ (Ton)		0.669670737	276.0709168	1471.795717	2442.565397	1855.311345	275.3949036	136.007419
GIIP (m^3^)		29289603.17	12074601979	64372399985	106831263920	81146345659	12045034974	5948599972

*Constant calculation parameters (ρCO_2_, B, and E) are identical for all wells.

Well-level results highlight the strong dependence of storage capacity on stratigraphic subdivision, net reservoir thickness, and effective porosity. In well OBA-2–3, multiple stacked Lower Safa subunits collectively provide the highest cumulative storage capacity (> 4,000 t), primarily driven by greater thickness and more favorable reservoir properties. Well OBA-D5 shows intermediate cumulative capacities (approximately 136–2,440 t) distributed across several Lower Safa subunits, while other intervals were classified as waste zones and excluded due to insufficient reservoir quality. In well OBA-D13, the exclusion of non-reservoir units results in a moderate cumulative storage capacity of (~ 960 tons).

Across all three wells, the calculated storage capacity varies systematically with differences in effective reservoir thickness, pore volume, and petrophysical properties (Tables 9, 10 and 11). A storage efficiency factor ranging between 0.05 and 0.2 was considered consistent with screening-level CCS assessments.

Comparative estimates across the three wells (Table 12) confirm that the Lower Safa Member consistently provides substantially greater storage capacity than the Upper Safa Member. Storage capacities vary among individual compartments according to differences in reservoir thickness, pore volume, and effective porosity. Table 13 summarizes the pressure-constrained storage capacities estimated for individual and aggregated fault-bounded compartments.

**Table 12:** Comparative CO_2_ storage capacity estimates for upper and lower Safa members in the obaiyed field.

Well / field	Reservoir member	Net reservoir units considered	Avg. effective porosity	(1 − Swi)	Pore volume	Estimated CO_2_ capacity	Storage potential “screening assessment”
			(–)		(m^3^)	(tons)	
OBA-2–3	Upper Safa	1	0.113	0.72	1.22 × 10^5^	0.51	Low
OBA-2–3	Lower Safa	3	0.147–0.179	0.44–0.73	1.86 × 10^8^	4088.1	Very high
OBA-D5	Upper Safa	1	0.087	0.79	1.45 × 10^5^	0.67	Low
OBA-D5	Lower Safa	5	0.082–0.112	0.59–0.93	2.46 × 10^8^	1471.8–2442.6	High
OBA-D13	Upper Safa	1	0.092	0.82	1.14 × 10^5^	0.55	Low
OBA-D13	Lower Safa	3	0.087–0.105	0.63–0.85	1.48 × 10^8^	961.4	Moderate
Western desert analog fields*	Jurassic–Lower Cretaceous	Field-scale	0.08–0.18	0.50–0.80	10^9^–10^11^	10–100 + Mt CO_2_	Proven / high

*GIIP is reported at reservoir conditions and is not used in the CO_2_ mass equation.

**Table 13:** Effective CO_2_ storage **capacity** in the Obaiyed Field, governed by pressure management rather than pore volume alone.

Reservoir member	Scale	ΔPmax (MPa)	Pressure-limited capacity (MtCO_2_)	Capacity limitation
Lower Safa	Single compartment	2 MPa	0.0006–0.0010	Strongly injectivity-limited
Lower Safa	Single compartment	5 MPa	0.0015–0.0024	Injectivity-limited
Lower Safa	Single compartment	10 MPa	0.0030–0.0045	Near volumetric limit
Lower Safa	Multi-compartment (aggregated)	2 MPa	0.002–0.004	Pressure-managed
Lower Safa	Multi-compartment (aggregated)	5 MPa	0.006–0.012	Balanced
Lower Safa	Multi-compartment (aggregated)	10 MPa	0.012–0.020	Volumetric-controlled
Upper Safa	Single interval	2–5 MPa	< 0.0001	Volumetrically limited

*Pressure-limited capacities represent conceptual estimates derived from assumed pressure constraints and are intended for comparative screening rather than operational design.

#### The spatial distribution of calculated CO_2_ storage capacity (MCO_2_)

**Fig. 29:**
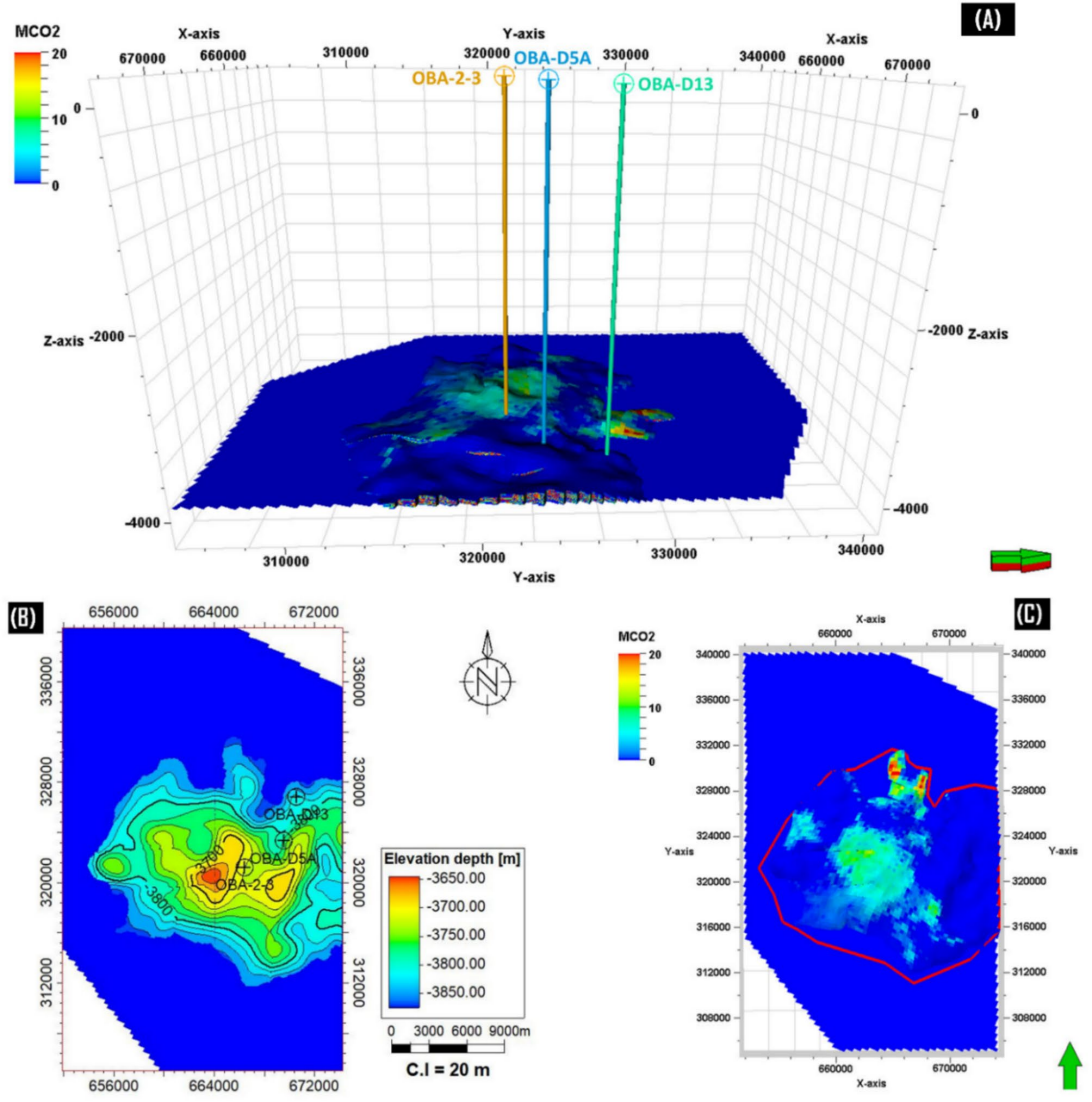
Spatial distribution of calculated CO_2_ storage capacity (MCO_2_) within the lower Safa member, obaiyed field. (**A**) Three-dimensional view showing the modeled MCO_2_ distribution at reservoir depth together with the vertical projections of wells OBA-2–3, OBA-D5A, and OBA-D13. (**B**) Plan-view structural depth map overlain by MCO_2_ contours (contour interval = 20 m), illustrating the correspondence between structural relief and variations in storage capacity. (**C**) Areal distribution of calculated MCO_2_ within the modeled field boundary, highlighting the concentration of storage potential in structurally compartmentalized zones.

The spatial distribution of calculated CO_2_ storage capacity (MCO_2_) across the Obaiyed Field (Fig. 29) reflects the structural framework and petrophysical variability documented in the preceding results. Three-dimensional visualization (Fig. 29A) indicates that higher MCO_2_ values are confined to localized zones within the Lower Safa reservoir, illustrating lateral variation in effective pore volume in the vicinity of the studied wells. Plan-view mapping (Fig. 29B) shows that increased storage capacity is preferentially associated with structurally deeper areas and intervals of greater reservoir thickness, pointing to a clear structural influence on volumetric capacity. The areal distribution within the modeled field boundary (Fig. 29C) further demonstrates that storage potential is concentrated within discrete fault-bounded compartments, while adjacent areas display markedly lower capacities. The calculated MCO_2_ distribution is spatially heterogeneous and follows the structural compartmentalization observed within the modeled reservoir.

#### Sensitivity analysis of storage capacity estimation

A sensitivity analysis was performed to evaluate the influence of the storage efficiency factor (E) on the estimated CO_2_ storage capacity of the Lower Safa Member (Table 14). Three efficiency scenarios (E = 0.4, 0.6, and 0.8) were evaluated to quantify the uncertainty associated with storage efficiency during screening-level volumetric assessment. As expected from the volumetric storage equation, the estimated storage capacity increases uniformly with increasing storage efficiency. Reducing the storage efficiency factor from 0.8 to 0.4 decreases the estimated storage capacity by approximately 50%, whereas intermediate efficiency values produce proportionally lower storage estimates.

**Table 14:** Sensitivity of estimated CO_2_ storage capacity to storage efficiency factor (E) for the lower Safa member.

Storage efficiency factor (E)	Estimated storage capacity (Mt CO_2_)
0.4	9.3
0.6	14.0
0.8	18.6

The base-case estimate assumes **E = 0.8** to represent effective pore-space utilization within structurally compartmentalized reservoirs characterized by favorable geological trapping conditions. The E value was selected as a **screening-level storage efficiency factor** for preliminary geological assessment rather than as a universal efficiency applicable to all CO_2_ storage projects. The evaluated efficiency range illustrates the uncertainty associated with this parameter and provides realistic upper and lower bounds for the estimated storage resource.

In short, the Lower Safa Member constantly maintains considerable storage potential throughout the tested efficiency range, supporting its selection as the principal interval for subsequent site-specific CO_2_ storage evaluation.

## Discussion

Structural compartmentalization emerges as the primary geological control on the CO_2_ storage potential of the Obaiyed Field, outweighing the influence of reservoir quality parameters alone. Although the Lower Safa Member exhibits higher effective porosity, greater net reservoir thickness, and lower irreducible water saturation than the Upper Safa Member, its superior storage performance results from the combined influence of reservoir architecture, effective pore volume, and fault-controlled compartmentalization. Comparable relationships have been reported from structurally segmented reservoirs, where storage efficiency is governed predominantly by trap geometry and seal integrity rather than permeability alone^2,3^.

The integrated workflow adopted in this study combines seismic interpretation, multimineral petrophysical evaluation using the Quanti Elan inversion approach, and fault-constrained three-dimensional geological modeling to provide a comprehensive geological screening framework for CO_2_ storage assessment in mature hydrocarbon fields. Rather than relying on a single reservoir attribute, this workflow integrates structural, stratigraphic, and petrophysical constraints to reduce geological uncertainty and improve confidence in storage evaluation. Similar integrated approaches have been widely recommended for preliminary CCS site screening because they provide a more realistic representation of reservoir heterogeneity and compartmentalization prior to dynamic simulation^38,45^.

### Reservoir controls on CO_2_ storage

Structural compartmentalization is a key control on storage performance in the Obaiyed Field because it simultaneously enhances containment and constrains injectivity. Fault-bounded compartments restrict lateral plume migration and improve structural confinement, whereas pressure build-up within individual compartments may reduce the amount of CO_2_ that can be injected under operational pressure limits. Consequently, the pressure-constrained sensitivity analysis indicates that effective storage capacity is governed by pressure management in addition to available pore volume, consistent with previous assessments of depleted hydrocarbon reservoirs^2,41^.

Although the estimated storage capacities of individual compartments are relatively modest, they are comparable to those reported for structurally segmented tight reservoirs. From a field-development perspective, multiple fault-bounded compartments can be evaluated collectively, allowing cumulative storage capacity to exceed that of individual compartments while maintaining structural isolation. Because similar Jurassic reservoir–seal successions and fault-controlled closures occur widely throughout the northwestern Western Desert, the geological characteristics identified in the Obaiyed Field may also provide a useful analogue for regional-scale geological screening of mature hydrocarbon provinces^3,38^.

The marked permeability contrast between the Lower Safa Member and the underlying Shifa Formation further supports the interpreted containment framework. The substantially lower permeability of the Shifa Formation is expected to restrict downward fluid migration, thereby enhancing vertical containment beneath the principal storage interval. Comparable tight reservoirs have been considered technically suitable for geological CO_2_ storage where injection is distributed among structurally isolated compartments and supported by appropriate pressure-management strategies^2,41^.

### Heterogeneity analysis

Core-derived permeability data indicate that the Safa reservoirs exhibit moderate-to-high heterogeneity, as reflected by both the coefficient of variation (CV) and the Dykstra–Parsons coefficient (VDP). The combined interpretation of these indices suggests that permeability contrasts are sufficiently pronounced to influence fluid-flow behavior at the compartment scale rather than across the reservoir as a whole. Similar relationships have been reported in heterogeneous clastic reservoirs, where localized high-permeability pathways coexist with intervals of restricted hydraulic communication^3,42^.

Within the Obaiyed Field, the observed heterogeneity is further reinforced by fault-controlled compartmentalization, resulting in spatially variable hydraulic connectivity. Such conditions are expected to influence CO_2_ plume migration and pressure propagation by promoting localized flow within structurally bounded compartments instead of uniform reservoir-scale displacement. This behavior is consistent with the geological architecture interpreted from the integrated structural and petrophysical models developed in this study.

### Fault-controlled compartmentalization

Fault-seal analysis indicates that structural compartmentalization exerts a fundamental control on fluid containment within the Lower Safa Member. Faults characterized predominantly by sand–shale juxtaposition (Faults 1, 12, 13, and 14) are interpreted as effective lateral seals or baffle-prone structures, consistent with the observed pressure compartmentalization and long-term hydrocarbon retention within the reservoir. Comparable sealing behavior has been documented in compartmentalized reservoirs where clay-rich fault rocks and favorable stratigraphic juxtaposition significantly reduce cross-fault fluid flow^35^.

In contrast, faults intersecting intervals of reduced reservoir continuity (Faults 6, 7, 8, 11, and 15) remain difficult to classify because local stratigraphic thinning and model-resolution limitations increase the uncertainty of fault-seal predictions. Faults 9 and 16 display mixed sand–sand and sand–shale juxtaposition, suggesting partially sealing behavior that may permit limited hydraulic communication while maintaining effective structural separation between adjacent compartments. This interpretation is consistent with the spatial distribution of juxtaposition patterns and the measured pressure differences observed across the Lower Safa reservoir.

Based on the geological evidence evaluated in this study, the partially sealing faults are not expected to compromise reservoir-scale containment under the assumed pressure conditions. Fault reactivation associated with elevated injection pressures remains an important geomechanical consideration during site-specific CCS design^4,46,47^. Consequently, geomechanical assessment and pressure-management strategies should accompany any future dynamic evaluation of CO_2_ injection scenarios.

### Fault containment behavior

Integration of fault permeability prediction with transmissibility analysis provides a more comprehensive assessment of fault behavior than lithological juxtaposition alone. The combined results suggest that most major faults within the Lower Safa Member are likely to function as effective or partially sealing barriers, enhancing structural compartmentalization and restricting lateral fluid migration. Similar fault-controlled flow behavior has been reported from structurally compartmentalized reservoirs, where permeability architecture and fault properties jointly govern fluid migration and storage performance^3,4^.

Although several faults exhibit intermediate transmissibility, these structures are interpreted as permitting limited hydraulic communication rather than acting as fully transmissive conduits. Consequently, the effectiveness of geological containment depends not only on fault-seal characteristics but also on maintaining injection pressures within safe operational limits. Previous studies have similarly identified the interaction between fault permeability, pressure evolution, and fault reactivation as one of the principal controls on long-term storage security in geological CO_2_ storage systems^46,47^.

The storage capacities presented in this study therefore represent geological screening estimates constrained by static structural and petrophysical conditions. Under operational injection scenarios, effective storage performance is expected to depend primarily on pressure evolution within individual fault-bounded compartments rather than on pore volume alone. Although dynamic reservoir simulation was beyond the scope of this work, integration of structural interpretation, core-derived petrophysical properties, and reservoir-pressure information provides a robust basis for preliminary assessment of CO_2_ storage potential. Future studies should incorporate coupled dynamic flow simulation and geomechanical analyses to evaluate injectivity, plume migration, pressure buildup, and long-term storage performance under field-specific injection conditions.

Fluid migration within the Lower Safa reservoir is interpreted to be governed predominantly by matrix flow through heterogeneous pore networks rather than fracture-dominated transport mechanisms. This interpretation is consistent with the relatively tight clastic character of the reservoir, where permeability contrasts and pore-scale connectivity exert greater control on pressure propagation than fracture-controlled flow. Consequently, reservoir heterogeneity and structural compartmentalization remain the dominant geological controls on CO_2_ migration and storage behavior within the Obaiyed Field^31,34,43,48^.

### Implications for CCS deployment in the Western Desert

Although this study focuses on the Obaiyed Field, the geological characteristics identified within the Jurassic Safa reservoirs have broader implications for geological CO_2_ storage throughout the Western Desert of Egypt. Structurally compartmentalized Jurassic reservoirs comparable to those of the Khatatba Formation occur widely across the Shushan, Matruh, and Abu Gharadig basins, where long-term hydrocarbon accumulation demonstrates the effectiveness of regional sealing systems and the persistence of structurally controlled hydrocarbon traps^6,7^.

Within the Obaiyed Field, the Lower Safa Member represents the principal storage interval because of its substantially greater effective pore volume and storage capacity relative to the Upper Safa Member. Nevertheless, the achievable injected CO_2_ volume is ultimately constrained by pressure evolution within individual fault-bounded compartments rather than by pore volume alone. Consequently, operational storage capacity is expected to depend on pressure management and injectivity in addition to volumetric capacity.

From a field-development perspective, structurally isolated compartments should be considered collectively rather than individually. Although storage capacity within a single compartment remains relatively limited, aggregation of multiple fault-bounded compartments combined with phased injection strategies provides a practical approach for increasing cumulative storage capacity while maintaining acceptable pressure conditions. Similar geological settings have been recognized in other depleted Jurassic–Lower Cretaceous reservoirs throughout the Western Desert, supporting the regional applicability of this storage concept^9–11^.

The present results further suggest that structural compartmentalization, traditionally regarded as a challenge for hydrocarbon production, may represent a favorable geological attribute for long-term CO_2_ storage. By restricting lateral plume migration and promoting pressure partitioning, fault-controlled compartments can improve containment efficiency while reducing the potential for uncontrolled reservoir-scale pressure propagation. These observations emphasize the importance of integrated geological screening workflows that combine structural interpretation, petrophysical characterization, and fault-seal evaluation to identify suitable storage sites within mature hydrocarbon provinces. Such integrated approaches are increasingly recognized as a key component of regional and global CCS deployment strategies^48^.

### Limitations and future work

The findings of this study should be interpreted within the context of several methodological limitations inherent to screening-level geological assessments. The evaluation integrates seismic interpretation, well-log–based petrophysical characterization, and three-dimensional static geological modeling to constrain reservoir architecture and structural compartmentalization; however, additional analyses are required before site-specific CO_2_ injection can be fully assessed.

The structural model was developed primarily from two-dimensional seismic data constrained by a limited number of wells. Although this approach is appropriate for regional geological screening, small-scale faults and subtle stratigraphic discontinuities may remain unresolved, introducing uncertainty in fault geometry, compartment boundaries, and local predictions of fluid migration.

Storage capacity was estimated using a static volumetric approach and therefore represents a first-order geological assessment rather than an operational storage forecast. Dynamic processes, including pressure evolution, injectivity, plume migration, and coupled geomechanical responses, were beyond the scope of the present investigation and should be evaluated through integrated dynamic reservoir simulation and geomechanical modeling.

Storage efficiency remains one of the principal sources of uncertainty in volumetric capacity estimation. Although relatively high storage efficiency may be achievable within structurally confined compartments, the effective injectable CO_2_ volume is ultimately governed by pressure buildup and injectivity constraints. To reduce uncertainty, the principal input parameters were constrained using measured core data together with geological interpretation. The resulting range of storage capacities demonstrates the sensitivity of volumetric estimates to reservoir heterogeneity and geological uncertainty.

Future investigations should incorporate higher-resolution seismic datasets, additional well control, coupled flow–geomechanical simulations, and fault-stability analyses to better quantify injectivity, pressure evolution, plume migration, and long-term containment under field-scale operating conditions.

Despite these limitations, the integrated workflow developed in this study demonstrates that combining structural interpretation, petrophysical characterization, and fault-seal analysis provides a practical geological screening approach for evaluating CO_2_ storage potential in mature, structurally compartmentalized hydrocarbon reservoirs.

## Conclusions

This study provides a geological screening assessment of CO_2_ storage potential within the Jurassic Safa reservoirs of the Obaiyed Field, Western Desert of Egypt. Integrated structural interpretation, petrophysical characterization, and three-dimensional geological modeling demonstrate that the Lower Safa Member represents the principal storage interval because of its greater effective pore volume, higher reservoir quality, and substantially larger storage capacity than the Upper Safa Member.

Fault-seal analysis further indicates that structural compartmentalization is a primary control on storage behavior. Sand–shale juxtaposition along major faults forms effective lateral seals, whereas partially sealing faults promote pressure partitioning while maintaining hydraulic communication between selected compartments. These geological characteristics enhance containment potential and support compartment-scale CO_2_ storage under appropriate pressure-management conditions.

Although individual fault-bounded compartments provide relatively modest storage capacity, aggregation of multiple structurally isolated compartments offers a practical pathway for increasing cumulative storage potential at the field scale. Because similar Jurassic reservoir–seal systems occur widely throughout the Western Desert, the geological framework developed in this study may also be applicable to regional screening of mature hydrocarbon provinces for geological CO_2_ storage.

Overall, this study demonstrates that integrating structural geology, petrophysical characterization, and fault-seal evaluation provides a robust geological basis for identifying secure CO_2_ storage opportunities in structurally compartmentalized hydrocarbon reservoirs and supports their evaluation as promising candidates for long-term geological CO_2_ storage.

## Supplementary Information

Supplementary Information 1.

Supplementary Information 2.

Supplementary Information 3.

## Data Availability

The datasets analyzed in this study were provided by the Egyptian General Petroleum Corporation (EGPC) through Badr El-Deen Petroleum Company (BAPETCO) and are subject to licensing and confidentiality restrictions. Consequently, the data are not publicly available. Access to the data may be granted by the corresponding author upon reasonable request and with prior permission from EGPC.
