# Supplementary material for: Fault-controlled reservoir compartmentalization as a control on CO2 storage potential in Jurassic Safa reservoirs, Obaiyed Field, Western Desert, Egypt
Source: Sci Rep. 2026 Aug 1;16:23722. doi: 10.1038/s41598-026-63564-2 (PMC13428752; doi:10.1038/s41598-026-63564-2)
Supplement: Supplementary file 1 — Supplementary Information 1. [file 41598_2026_63564_MOESM1_ESM.docx]

**Fault thickness distributions**

The three-dimensional fault thickness models were generated during structural modeling to quantify variations in fault thickness along the interpreted fault surfaces. These models were used as supporting input for the integrated fault seal evaluation, including fault rock characterization, fault permeability prediction, and transmissibility analysis. Although fault thickness is not interpreted independently as a direct indicator of sealing capacity, it provides important geometric information that complements lithological juxtaposition and fault property analyses used to assess reservoir compartmentalization and potential CO₂ containment.

**Effective Cross fault permeability**

The three-dimensional effective fault permeability models were generated to quantify spatial variations in hydraulic properties along the interpreted fault surfaces within the Lower Safa reservoir. Fault permeability was estimated using the integrated fault-seal workflow and provides a quantitative measure of the potential for cross-fault fluid flow. The modeled permeability distributions were used together with fault juxtaposition analysis, fault rock classification, and transmissibility calculations to classify faults as **sealing**, **partially sealing**, or **uncertain**. These supplementary figures provide the detailed permeability models supporting the summarized fault classification presented in the main manuscript.

**
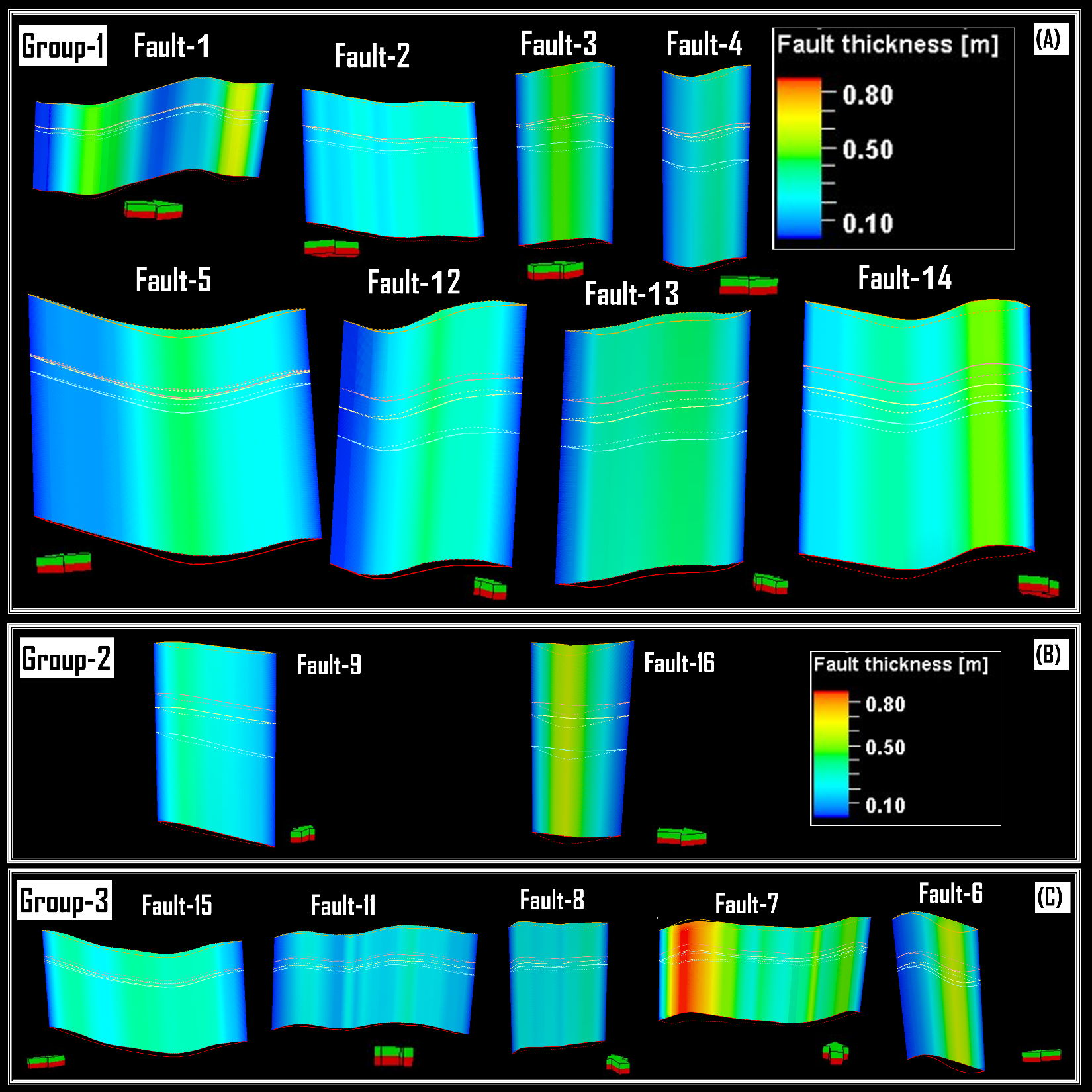
**

**Supplementary Figure S1. Three-dimensional fault thickness models used for fault seal analysis of the Lower Safa Member.** Fault thickness distributions were generated for the sixteen interpreted faults and grouped according to their structural behavior. (A) Group 1 includes faults interpreted as predominantly sealing. (B) Group 2 comprises partially sealing faults. (C) Group 3 includes faults exhibiting uncertain sealing behavior. Colors represent modeled fault thickness (m), which forms one of the input parameters used for fault rock characterization, fault permeability estimation, and transmissibility analysis.


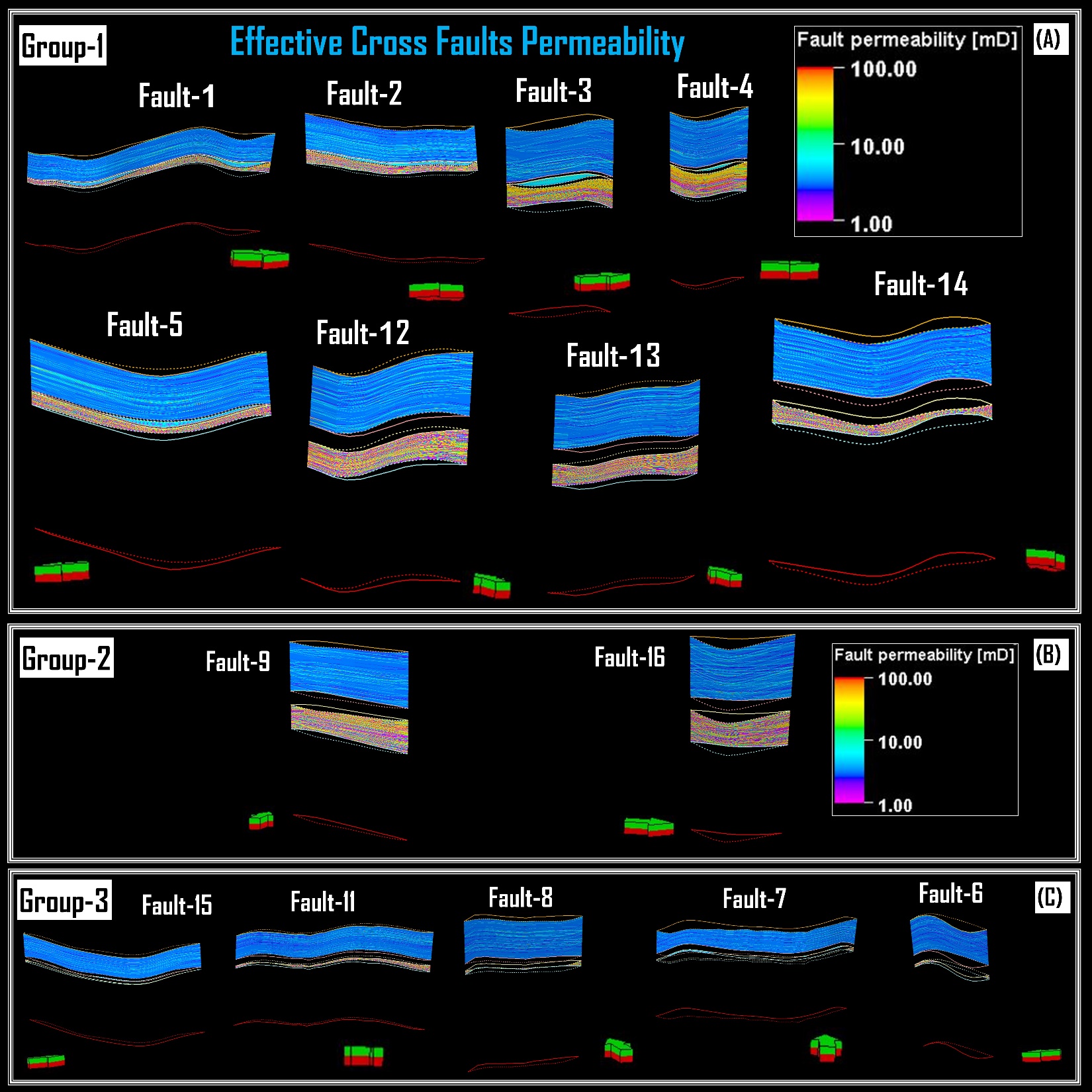


**Supplementary Figure S2. Three-dimensional effective fault permeability models for the interpreted faults within the Lower Safa Member.** Faults are grouped according to their interpreted sealing behavior: **(A)** Group 1—predominantly sealing faults (Faults 1, 2, 3, 4, 5, 12, 13, and 14); **(B)** Group 2—partially sealing faults (Faults 9 and 16); and **(C)** Group 3—faults with uncertain sealing behavior (Faults 6, 7, 8, 11, and 15). Colors represent modeled effective fault permeability (mD), ranging from low-permeability sealing zones to relatively higher-permeability intervals. These models were integrated with lithological juxtaposition, fault rock characterization, and transmissibility analyses to evaluate fault-controlled compartmentalization and potential CO₂ containment.
